# Training, environmental and nutritional practices in indoor cycling: an explorative cross-sectional questionnaire analysis

**DOI:** 10.3389/fspor.2024.1433368

**Published:** 2024-10-11

**Authors:** W. M. Peeters, A. H. Coussens, I. Spears, O. Jeffries

**Affiliations:** School of Biomedical, Nutrition and Sport Sciences, Newcastle University, Newcastle-upon-Tyne, United Kingdom

**Keywords:** exercise, cycling, thermoregulation, nutrition, technology

## Abstract

**Introduction:**

Indoor cycling at home has grown rapidly in recent years facilitated by advances in technology and gamification. However, there is limited data on individual's training practices when cycling indoors.

**Methods:**

Using a single-time point, cross-sectional questionnaire, we gathered information on equipment, environmental considerations, training practices and nutrition during indoor cycling.

**Results:**

Following 492 responses, external variables (weather; 88.4%, lack of daylight; 56.3%), time efficiency (81.9%) and general fitness (70.9%) were most frequently cited as reasons to engage in indoor cycling. “Smart” turbo trainers linked to a mixed-reality cycling software were most frequently reported in equipment set-up. 78% of participants attempted to control temperature with 96% of these participants using at least 1 fan to control airflow. The volume of indoor training differed between seasons (winter: 6h10 ± 3 h 30, summer 2h52 ± 2h57, *p* < 0.001), and structured (61.9%) and unstructured work-outs (64.7%) were completed more than once a week with fewer participants engaging in competitive/racing events (20.9%). 98% percent of participants consumed fluids with an average fluid intake of 0.74 ± 0.28 L/h. Dependent on type of work-out, participants reported less planning of carbohydrate and protein intake during short-duration work-outs (∼40%–60%) relative to longer-duration work-outs (∼56%–80%). Caffeine use was the most frequently reported ergogenic aid.

**Conclusion:**

Together we report indoor cycling practices with respect to training equipment, considerations of environmental and nutritional strategies and training habits. Our findings could be used to support the development of future research and indoor training guidelines.

## 1 Introduction

Indoor cycling using online mixed-reality training platforms increased dramatically coinciding with COVID-19 lockdown events around the world (1, 2). Advances in technology have enabled recording of cycling power measured via smart trainers, portable power meter devices, or indoor specific training bikes. When combined with online 2-D or 3-D games or training simulations, which facilitates the at-home indoor cycling experience, it can be a powerfully immersive tool to improve exercise adherence (3, 4). Moreover, during COVID-19 when outdoor races were restricted, indoor cycling facilitated racing events for professional cyclists by staging a virtual Tour de France (2) and one day classic [Tour of Flanders (5)], culminating in the first UCI-regulated indoor cycling world championships (6) whilst also triathlon has embraced virtual reality within some competition formats (7). Further, talent identification programs using home-based, mixed-reality cycling platforms have emerged since 2016, with riders achieving professional road cycling contracts (8).

Indoor cycling can also fulfil a number of roles in non-elite populations. Clear health benefits exist for indoor cycling favoring reductions in body weight and improvements in cardio-respiratory fitness (9, 10) and protocols within software to monitor training adaptations, such as a 20-min functional threshold power test, reduce the need to access specialist equipment. It can serve as a tool during athlete's recovering from injury to remain active and can be specifically used for controlled high intensity efforts eliminating the risks associated with road use and traffic. Indoor cycling may also be favored when environmental conditions are poor such as rain, snow, ice, wind or extremes of heat and cold (11) whilst recent gamification via online mixed-reality platforms can further incentivize, motivate and engage individuals to make a training program more enjoyable (12). Additionally, home-based indoor cycling creates a controlled environment whereby individuals can practice nutritional strategies, for example carbohydrate feeding or optimal hydration, amongst others, to prepare for competitive events. However, to date there is limited understanding regarding the preferential use of indoor cycling and the effect on training habits with only data on nutritional practices being investigated (13).

Arguably indoor cycling may elicit a comparable training stress to outdoor cycling (14–19); however, greater levels of perceived effort have been described for indoor cycling (20), and physiological stress may be reduced meaning that training-induced stress and thus training adaptations may differ (21). These differences may be due to the stationary nature of indoor training thereby increasing environmental stress (air temperature, humidity, air movements), variation in road surface, enhanced visual exploration of outdoor environment (predictable vs. unpredictable attentional effort (22), and greater demands for balance and proprioception when not on a stationary bike (23).

It is reasonable to assume that there is variation in the way individuals engage with indoor cycling which may reflect geographical location, environmental constraints, training and fitness goals, finances and knowledge of optimal practice. Therefore, the aims of this questionnaire are to capture indoor training habits, to identify equipment set-up, environmental control, exercise training and nutrition. The aims of this questionnaire are to facilitate the development of optimal guidelines for home-based indoor cycling exercise and a growing awareness of indoor cycling habits and to identify areas for future scientific research experiments.

## 2 Methods

### 2.1 Participants and study design

This study used a single time point cross-sectional questionnaire design, emphasizing four themes related to indoor cycling: (i) equipment set-up, (ii) environmental set-up, (iii) training habits and, (iv) nutritional strategies. The study was approved by the local university Ethics Committee (Ref: 26899/2022). A purposeful sampling approach was employed as participants were eligible if they participated in indoor cycling and were aged 18 years and older. Participants were recruited via advertisement on social media and online indoor cycling platforms. Advertisements were placed on Strava™ community groups associated with known virtual cycling software (E.g., Zwift™, Wahoo™, Fulgaz™ etc.) and via Facebook cycling groups. The questionnaire was accessible to participants from January 2023 until July 2023. Participants had 30 days to complete the questionnaire once they started it. After providing informed consent, demographical information was gathered including age, gender, self-reported weight, ethnicity, and current country of residence. Additionally, current physical and cycling activity status was gathered by self-reported level of cycling (e.g., recreational, competitive, professional), exercise and cycling status (h/week), last known measurement of functional threshold power (FTP), as well as selecting primary reasons for engaging in indoor cycling.

### 2.2 Questionnaire design

Participants were provided with an online link to access the questionnaire (Qualtrics, January 2022, Provo) which was available in English. Prior to recruitment, two members of the research department, who were not involved in the study and had varying levels of experience with indoor cycling, piloted the questionnaire twice. Their feedback was incorporated before the questionnaire was released to participants. Prior to demographic information and informed consent being gathered, participants were informed that the questionnaire would be split into two parts to provide the questionnaire with structure, Part A and Part B. Part A asked questions about equipment set-up and environmental control. Part B asked questions about training habits and nutritional practices during indoor cycling. Participants were asked to complete both Part A and Part B of the questionnaire and were made aware that each section would take between 5 and 10 min to complete but to limit survey fatigue, participants were given the option to terminate the survey after completing Part A. The questionnaire consisted of maximally 110 questions and a link to the full questionnaire is provided in Supplementary Material S1.

Part A consisted of two blocks of questions, presented in a fixed order. The first block asked questions about equipment set-up including: (i) type of virtual software used, (ii) type of turbo-trainer used, (iii) type and positioning of device used to display virtual software and (iv) any additional accessories used to enhance the cycling experience. The second block asked questions about environmental control, including: (i) practices to control the temperature of the room in both summer and winter, (ii) practices to control body temperature and (iii) specific details on the use and positioning of fans to support environmental control, including estimated rotor diameter and distance from the bike. In part B—block three, questions were asked about training practices including: (i) frequency of exercise sessions of different durations (short: > 1 h, long < 1 h), (ii) seasonal differences (winter and summer) and time of day of exercise sessions and (iii) frequency of type of exercise session (race event, structured session e.g., intervals, unstructured sessions e.g., leisure/social group ride). In part B—block four, questions asked participants about nutritional practices including: (i) fluid intake, (ii) deliberate and intentional intake of carbohydrate and protein intake with respect timing (before, during and after exercise), different durations (> 1 h and < 1 h) and type of exercise session (race, structured, unstructured) and (iii) the use of nutritional supplements in different types of exercise sessions.

Several questions included an option “other” followed by an open text-box where participants could provide written details. At the end of each block, an open question was included if the participant wished to provide any additional information regarding their indoor cycling practices.

### 2.3 Data analysis

All data were exported to Microsoft Excel and two researchers (WMP, AHC) independently screened for completeness. Participants with any partial data were removed from the study. Although, if participants had only completed Part A, but not Part B, their data was included in the study. Where disagreement on data exclusion existed, researchers would discuss and where necessary a third researcher (OJ) was involved for a final decision.

To establish the position of fan placement, participants completed three fixed-answer questions to indicate distance away from handlebar, height and orientation around the bike. By integrating these three questions into one location, Cartesian coordinate data were retrieved from the server and processed using MATLAB (R2022a). The base of the front wheel was set to the origin (0,0,0) and medio-lateral, antero-posterior and vertical-axes were mapped to the x-, y- and z-axes, respectively. The 3-dimensional coordinates were then projected onto frontal, sagittal and horizontal planes and frequencies at each location in the planar grid were determined. To account for variability and uncertainty in the fixed-option answers to the true position of the fan, these frequencies were then interpolated bilinearly using a 2-dimensional Gaussian smoothing kernel intervals of 0.1 m and standard deviation of 0.5 to create planar frequency plots which were then mapped into the 3-dimensional representation in frontal, sagittal and horizontal planes using Unity3d. Fan air speed was determined by multiplying blade diameter by approximated fan speed and converted into appropriate units (m/s).

The frequency in number of response and percentage data were reported for the majority of questions in both Part A and Part B. Other data were reported as a specific value, including questions on duration of cycling, and nutritional consumption. Where relevant, parametric data (e.g., rate of perceived exertion between different types of indoor sessions or average hours completing indoor cycling across seasons) was analyzed in GraphPad Prism 9 using a one-way ANOVA with Tukey's post-hoc for multiple comparison where a significant effect (*p* < 0.05) was present. Relationships between fitness and participation in exercise were assessed using Pearson correlation or Kendall's tau for categorical data. Any open-text questions were analyzed employing thematic analysis, as responses were coded and grouped after independent analysis by two researchers.

## 3 Results

### 3.1 Demographics

**Figure 1:**
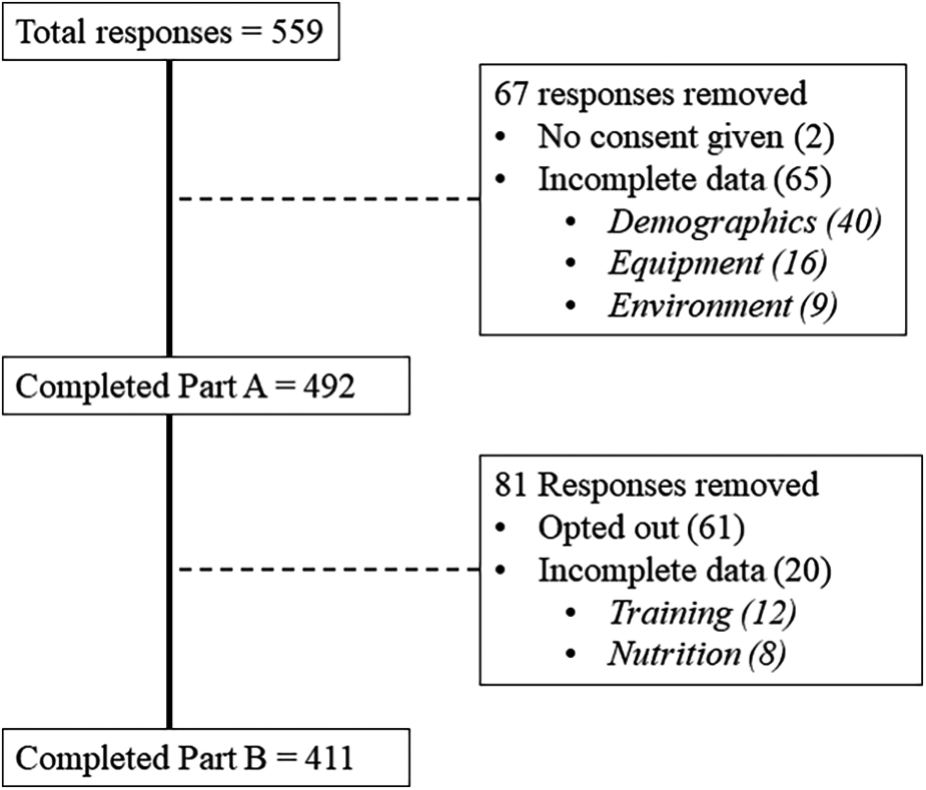
Flow chart of responses and reasons for drop-outs in part A and B of the questionnaire. Following completion of Part A, participants were asked if they wished to continue.

**Figure 2:**
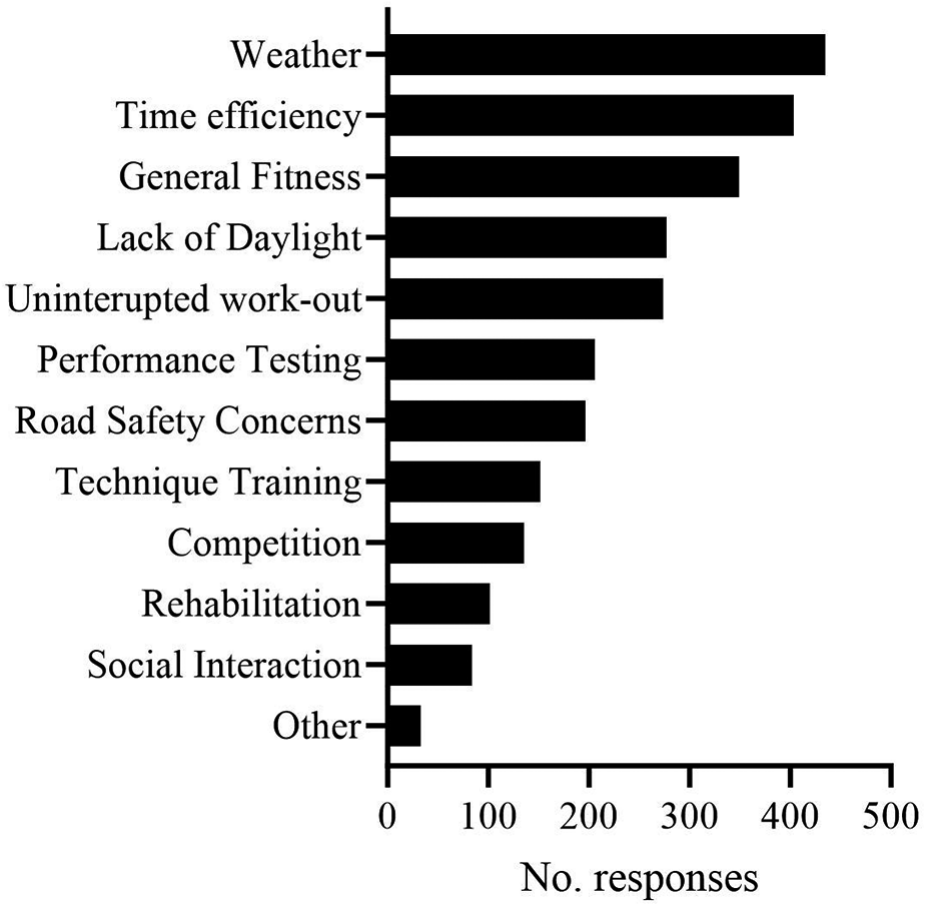
Reasons to engage in indoor cycling. Participants were allowed to select all that apply.

A flowchart of participant inclusion and exclusion is presented in Figure 1. A total of 492 people completed part A, whereas 411 completed the full questionnaire. Demographics reported in Table 1 are based on completion of part A. Self-reported average weekly exercise, all forms of cycling and indoor cycling hours were 10.2 ± 5.2, 8.2 ± 4.5 and 4.5 ± 3.8 h/w respectively. Participants identified themselves as recreational/amateur non-competitor (69.7%), amateur competitor (27.4%) or national level competitor or above (2.8%). Self-reported functional threshold power was 240 ± 55 W (3.1 ± 0.8 W/kg), which showed a moderate positive correlation with total exercise hours (*r* = 0.316, *p* < 0.001) but not indoor cycling hours (*r* = −0.044, *p* = 0.34) and moderate negative correlation with age (*r* = −0.425, *p* < 0.001). Weather, time efficiency and general fitness were reported as most common reasons to engage in indoor cycling (Figure 2).

**Table 1:** Demographic information.

Demographics	N	Mean/percentage	SD
Age (yr)	492	46.2	13.8
Body Mass (kg)		78.2	13.3
Gender			
Male	437	88.8%	
Female	55	11.2%	
Ethnicity identity			
White	445	92.3%	
Asian	22	4.5%	
Multi-ethnicity	10	2.0%	
Other ethnic groups	10	2.0%	
Black	3	0.6%	
Prefer not to say	2	0.4%	
Country of residence			
United Kingdom	213	43.3%	
Other	98	19.9%	
United States of America	66	13.4%	
Australia	29	5.9%	
Germany	24	4.9%	
Canada	20	4.1%	
Netherlands	17	3.5%	
Republic of Ireland	14	2.8%	
France	11	2.2%	

### 3.2 Equipment set-up

Most participants reported to use a direct drive turbo trainer (71.6%), followed by a smart-bike (17.4%), wheel-on turbo trainer (9.6%) and rollers (1.4%). Only 2.3% of participants reported not to use a virtual cycling software. Those using a virtual cycling software indicated that software was displayed predominantly on their laptop (32.1%), TV screen (26%) and tablet (22.9%), whilst fewer used their mobile phone (9.6%), PC/Desktop monitor (6%) or projector (1.2%) as display. Selecting the location of the display was mainly decided due to space/room constraints (45.3%), eye-sight (25.6%) and performance optimization (13.8%), whilst a fraction of participants indicated to position their display for neck-strengthening training (3.2%). Other reasons for the location of display suggested in open-text comments included, built-in screens on smart bikes, tablet holders, and using designated stands within reach. To aid in gamification 6.7% of respondents reported the use of a rocker plate, whilst 27.6% used equipment to alter simulated climbing gradients. Open-text comments (*n* = 128) to report additional habits of equipment set-up were themed around the use of multiple devices to watch entertainment/live-sport on a secondary screen or using apps for social communication. Mechanisms of cooling were mentioned; however this item was addressed in the next block. Additionally, using earphones or speakers to play music was frequently addressed as part of the equipment set-up.

### 3.3 Environmental control

When cycling indoors, most participants (68.3%) said they controlled temperature in both summer and winter months. 14.8% of participants indicated that they controlled temperature only during summer months, 5.3% only in winter months, and 11.4% reported that they did not attempt to control temperature at all. Temperature control in summer or winter was achieved by the use of a fan (90% and 75% respectively), opening windows (63% vs. 43%) and using air conditioning (17% vs. 6%). Only 1% of people moved outdoors in the summer and 12% used central heating during winter. One person stated that they used a dehumidifier.

**Figure 3:**
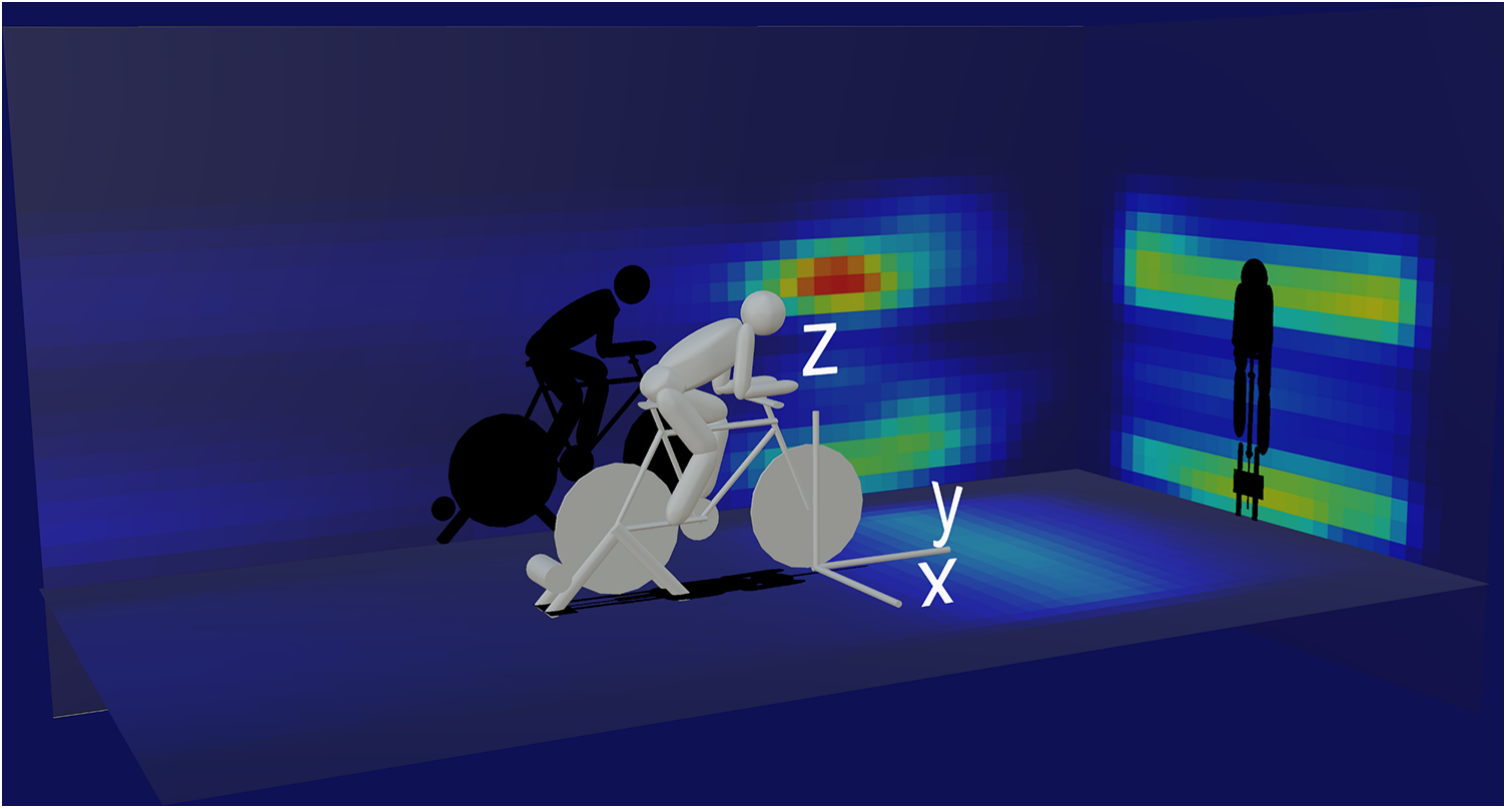
3-dimensional heat-map visualization of self-reported fan position to aid in thermal cooling during indoor cycling.

Control of air flow was achieved by using one fan (73%), two fans (20%) and three or more (3%). Estimated fan blade diameter (length end to end) was reported as 27 ± 12 cm with 18% reporting blade diameter between 10 and 20 cm and 23% reporting blade diameter between 20 and 30 cm. Fan speed on a three-point scale was reported as slow (16%), medium (37%) or high (41%). Placement of the fan was between 0.5–1 m (46%), 1–1.5 m (34%), 1.5–2 m (11%), greater than 2 m (>2%). Fan position was generally either placed directly in front of the cyclist (40%) to the front left (36%) or front right (29%) (Figure 3). Fan height was favored at face height (40%) or ground level (35%) (Figure 3). 57.8% of participants identified their fan position as optimal, whilst 28.9% and 11.8% identified space constraints and equipment constraints respectively as reason for not having the fan in the most optimal position.

When asked whether participants had ever attempted to raise or lower body temperature prior to indoor cycling, 66% of participants stated that they had never attempted to lower body temperature and 71% stated that they had never attempted to raise body temperature. Those who did attempt to lower body temperature typically used: cold air exposure (17%), cold fluid/ice ingestion (17%), cold water immersion (3%) or an ice vest (2%). To raise body temperature participants used: extra clothing (3%), warm air exposure (4%), hot water immersion (2%), or hot fluid ingestion (2%). When asked whether they had ever used indoor cycling to improve heat tolerance in preparation for a hot climate, 16% indicated that they had used this approach.

When asked what clothing participants typically wore during indoor cycling, 42% wore shorts only, 53% shorts and base layer, 15% shorts, base layer and short sleeve jersey and 2% shorts, base layer and long sleeve jersey.

Further open-text comments (*n* = 59) were themed around methods of sweat removal (towel/hair-bands) and adjusting temperature control within a session (removal of layers, intensity/heart rate-controlled fan speed).

### 3.4 Training practices

Reported average duration of an indoor cycling session was 72 ± 25 min, whilst the longest reported indoor cycling session conducted was 4 h ± 48 min. The average hours completed per week as indoor cycling was significantly different with 6 h 10 min ± 3 h 30 min during winter months and 2 h 52 min ± 2 h 57 min during the summer months (*p* < 0.001). During winter months individuals selected morning (31%), afternoon (16.2%) or evening (52.8%) to complete indoor cycling. During the summer months this shifted with participants selecting morning (43.2%), afternoon (12.8%) and evening (44%).

**Figure 4:**
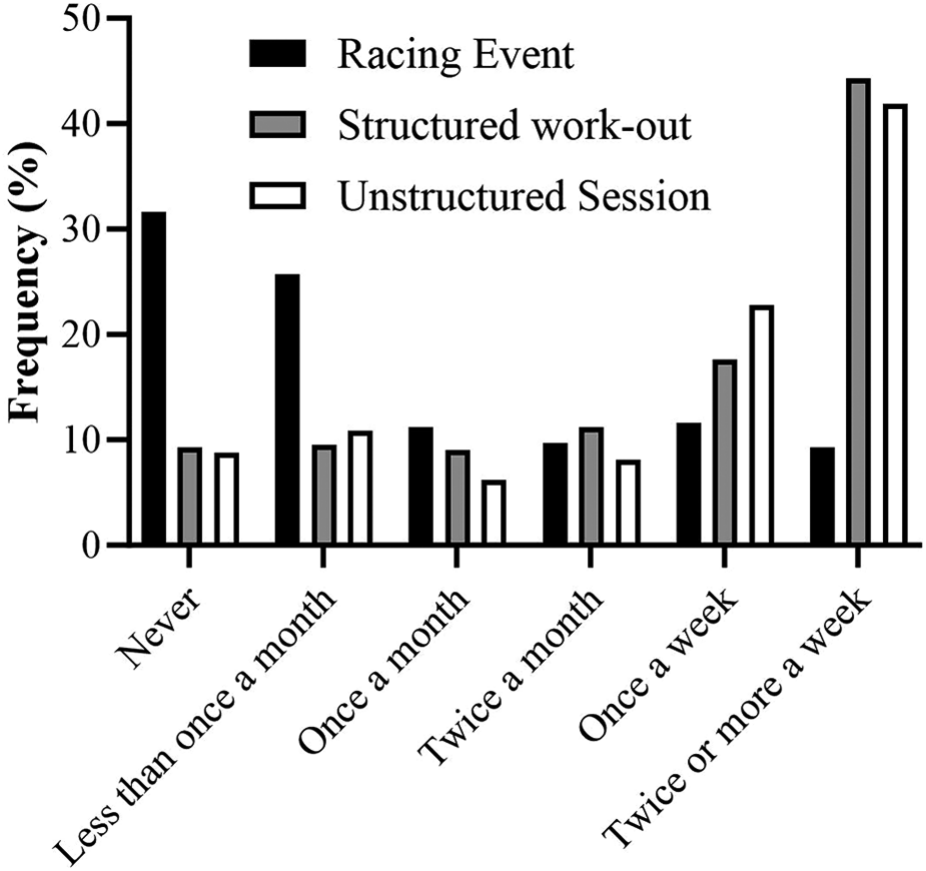
Frequency data for engaging in different types of indoor cycling sessions.

Structured work-outs and unstructured cycling sessions were reported to be completed most frequently, with 70% and 64.7% of respondents completing such sessions once a week or more respectively. In comparison, the frequency of completing racing events more than once a week was 20.8% whilst 31.9% reported to have never engaged in online racing events, with 9.2% and 8.7% having never engaged in structured work-outs and unstructured sessions respectively (Figure 4). Rate of perceived exertion (1–10 scale) was significantly different between all the types of sessions (Racing event: 8.6 ± 1.5, Structured work-out: 7.5 ± 1.5, Unstructured session: 5.5 ± 1.8, *p* < 0.001). Ranking the frequency of engaging in racing events and structured work-outs with FTP, there was a positive correlation with FTP (Race: *τ* = 0.179, *p* < 0.001, Structured: *τ* = 0.095, *p* = 0.009), and a negative correlation between unstructured sessions and FTP (*τ* = −0.125, *p* < 0.001).

Further open-text comments (*n* = 48) were themed around training practices focused on: “Zone 2” training, which likely refers to training at the exercise intensity below the lactate threshold, social group/club rides, gamification and completing challenges, and hill climb practice.

### 3.5 Nutritional practices

Fluid consumption was incorporated during indoor cycling sessions in 98% of participants, with an average hourly intake rate of 0.74 ± 0.28 L/h. 41% of participants indicated that they exclusively consumed plain water, 18.7% added a combination of electrolytes and carbohydrates with 22.4% exclusively adding electrolytes and 4.4% exclusively adding carbohydrates. Other open-text comments reported adding flavoring or that the content was dependent on the type of session.

Practices around carbohydrate and protein intake for different types and durations of cycling sessions are reported in Table 2. Overall, the proportion of respondents who have some form (before, during or after) of deliberately planning their nutrition was higher for longer-duration sessions (range based on type of session: 56%–80%) compared to short-duration sessions (range based on type of session: 40%–60%). Deliberate nutritional planning was reported to be more prevalent in racing events and structured work-outs compared to unstructured sessions and was most frequently reported for carbohydrate intake during exercise and protein intake after exercise.

**Table 2:** Self-reported prevalence (*n* = 411) of deliberate and intentional practices of carbohydrate and protein intake around different types and durations of indoor cycling.

	Before	During	After	No Plan	N/A	Before (g/kg BW)	During CHO (g/h) PRO (g/kg BW)	After (g/kg BW)
*Short-duration (<1 h)*								
*Racing Event*								
Carbohydrates	133 (44.8%)	80 (26.9%)	73 (24.6%)	108 (36.4%)	109	0.5 ± 0.3	33.7 ± 16.4	0.5 ± 0.3
Protein	14 (4.9%)	6 (2.1%)	137 (47.9%)	138 (51.7%)	114	0.33 ± 0.18	0.23 ± 0.14	0.35 ± 0.13
*Structured work-out*								
Carbohydrates	126 (34.1%)	75 (20.3%)	85 (23.0%)	157 (42.5%)	38	0.5 ± 0.2	34.4 ± 18.6	0.5 ± 0.3
Protein	11 (3.1%)	4 (1.1%)	166 (46.8%)	183 (51.5%)	43	0.31 ± 0.12	0.17 ± 0.05	0.35 ± 0.14
*Unstructured session*								
Carbohydrates	69 (18.9%)	55 (15.1%)	69 (18.9%)	213 (58.4%)	41	0.5 ± 0.3	33.5 ± 19.1	0.5 ± 0.3
Protein	13 (3.7%)	5 (1.4%)	132 (37.3%)	312 (60.2%)	46	0.31 ± 0.12	0.31 ± 0.18	0.34 ± 0.15
*Long-duration (>1 h)*								
*Racing Event*								
Carbohydrates	148 (54.6%)	177 (65.3%)	79 (29.2%)	53 (19.6%)	139	0.6 ± 0.3	38.7 ± 18.4	0.6 ± 0.4
Protein	19 (6.6%)	13 (4.5%)	162 (55.9%)	112 (38.6%)	111	0.32 ± 0.14	0.29 ± 0.18	0.37 ± 0.14
*Structured work-out*								
Carbohydrates	174 (48.5%)	221 (61.6%)	111 (30.9%)	74 (20.6%)	49	0.6 ± 0.3	38.5 ± 18.7	0.6 ± 0.3
Protein	25 (7.1%)	12 (3.4%)	208 (59.3%)	131 (37.3%)	48	0.33 ± 0.13	0.30 ± 0.18	0.37 ± 0.14
*Unstructured session*								
Carbohydrates	128 (34.6%)	210 (56.8%)	106 (28.6%)	100 (20.6%)	37	0.6 ± 0.3	36.5 ± 19.4	0.6 ± 0.3
Protein	26 (7.2%)	17 (4.7%)	185 (51.4%)	155 (43.1%)	40	0.34 ± 0.12	0.32 ± 0.18	0.36 ± 0.14

N/A; not applicable refers to a response “never engaged in such session”. The percentages of participants indicating to not have a plan was based on responses excluding those who never engaged in such session.

**Figure 5:**
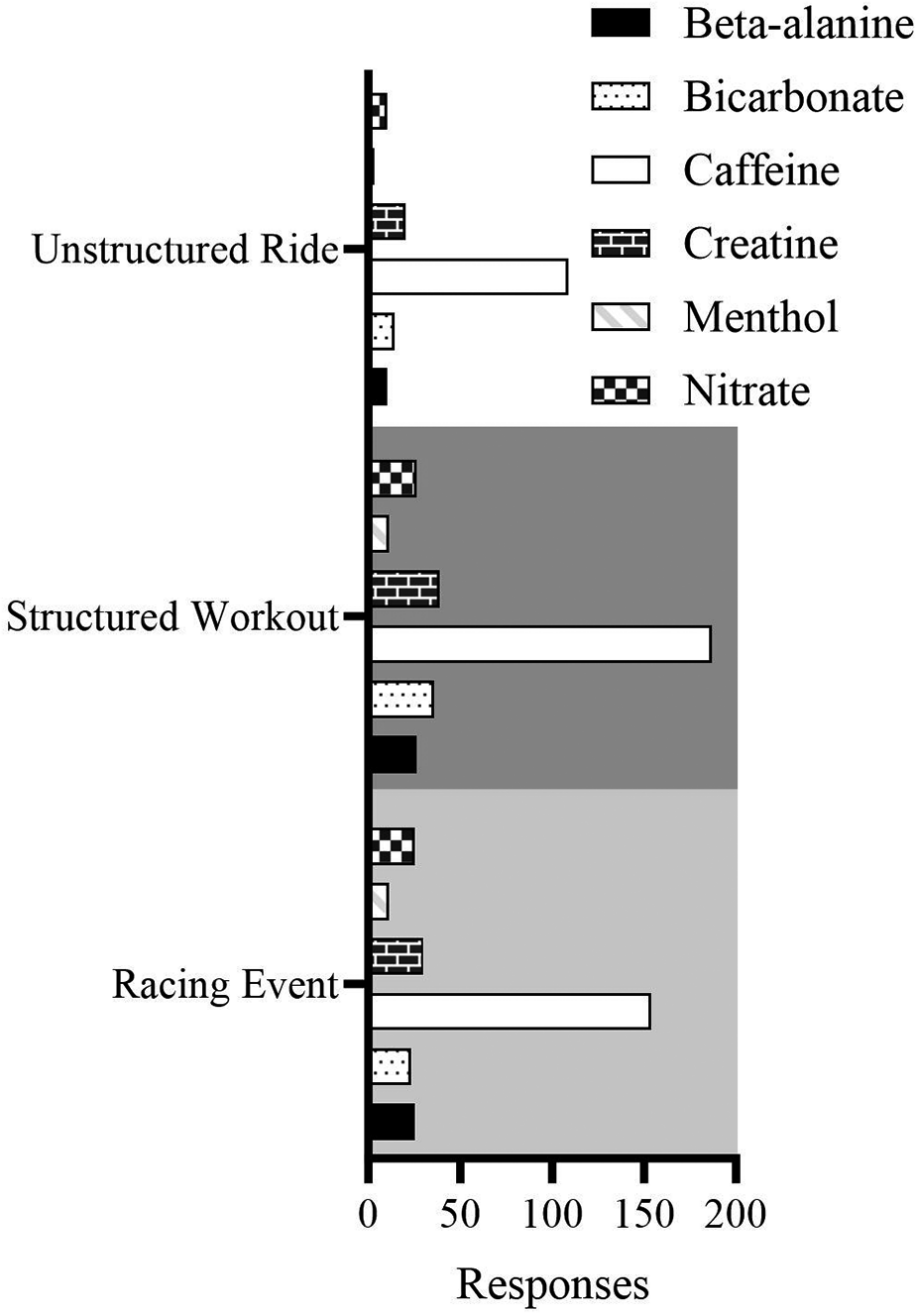
Frequency data on the use of nutritional ergogenic aids to complete different types of indoor cycling sessions.

Most respondents (63%) indicated to have used caffeine as an ergogenic aid or supplement; however, the proportion of participants who reported to have never used other (beta-alanine, bi-carbonate, creatine, nitrate and menthol) ergogenic aids was larger than 88% (Figure 5).

Further open-text comments (*n* = 60) were themed around the sources of nutrition, mostly related to consumption of whole-foods or sport nutrition products. Additionally, respondents commented on completing indoor cycling session in a fasted state for unstructured work-outs (e.g., “zone 2”).

## 4 Discussion

The recent prevalence in indoor cycling has resulted from advances in technology both in equipment and immersive online platforms. The major findings of the present cross-sectional questionnaire are that, overall 98% of the individuals sampled used mixed-reality software, 96% of individuals controlled the thermal stress they experience by use of at least one fan and 98% consumed fluids during cycling. Time efficiency was reported as the predominant (>80%) reason to select indoor cycling vs. outdoor cycling. However, a more variable picture existed around practices of training (seasonal variation, time of day, type of session) and nutrition where between 40%–80% of participants deliberately planned their carbohydrate or protein intake during indoor cycling which varied depending on the type and duration of sessions. It should be noted that the results are collected from a cohort that was predominantly middle-aged males of white ethnicity from English-speaking countries, thus extrapolation of these results to other cycling cohorts should be made with caution.

### 4.1 Equipment set-up

Most participants (∼72%) reported to use a direct drive trainer. Although variability in accuracy will exist between brands and models, there is evidence that direct drive models provide reliable and valid power readings (24, 25), whereas wheel-on turbo trainers are less accurate (26). However, the direct drive trainer analysed by Zadow et al. (24) showed larger positive bias for 100–200 W (LoA: 4.5%) and 750–999 W (LoA: 13%) compared to a gold-standard CALRIG device. Therefore, caution is required when performance monitoring is completed within these exercise ranges e.g., lactate thresholds or maximal sprint performance. Conversely, the direct drive trainer tested by Morais et al. (25) only reported the accuracy between 100–270 W and compared the values against a pedal power meter that was shown not to be reliable when compared to the Lode Excalibur (27). There are no scientific studies examining the reliability and validity of smart bikes used by ∼17% of the individuals surveyed. 97% of individuals used online software for their indoor training, providing clear evidence of the increasing popularity of online immersive platforms, however bias in recruitment platforms might have overestimated this outcome. The adoption of emerging technologies to facilitate realism in indoor cycling such as rocker plates which permit lateral movements and climbing simulators that increase the gradient of the bicycle were relatively limited. The current discussion in the literature, regarding whether indoor and outdoor cycling offer a comparable training stress (14–16, 18); or in fact provide a greater perceived effort (20), and reduced cardiovascular (17) and neuromuscular stress (21) indoors, mean that further research should be directed towards understanding how technology may further mimic the physical, perceptual and physiological demands of outdoor cycling. These technologies may not only facilitate a greater physical adaption to indoor cycling but may be beneficial for training adherence.

### 4.2 Environmental control

A rise in core body temperature is a natural result of exercise in a warm, environment where convective air flow is limited (28), typified by indoor conditions. Even in relatively cool conditions where air temperature is between 4 and 8 ^o^C body core temperature can rise by >1 ^o^C during a 60 min intensive cycling task, with greater increases as ambient room temperature increases (29, 30). It is well established that increased body core temperature is associated with a decrease in exercise performance; therefore, strategies to expand or limit heat storage capacity are essential to improve indoor cycling performance. Modulation of air temperature, enhancing convective air flow or controlling changes in body temperature, are the primary strategies that can be utilized to offset rises in body temperature and heart rate when training indoors and enhance thermal comfort (31). Indeed, a recent SWOT analysis on virtual training identified potential health risks around physiological strain derived from limited air flow and dehydration (11). However, generally in the sample population examined, there was good awareness of strategies to modulate environmental temperature with ∼80% of respondents confirming that they took some approach to altering temperature in summer and winter months. Typically, this was achieved using a fan (∼90%), opening windows (∼63%) or using air conditioning (∼6%). In winter months the use of these strategies was reduced which may reflect the cooler ambient temperatures. Air flow across the body facilitates convective heat exchange and aids evaporative heat loss (28), with a progressive reduction in exercise performance observed with decreasing air velocity (32). In this questionnaire, ∼70% of participants reported using on average ∼1 fan, placed approximately 1–1.5 m away, largely at the front, at face height or ground level, and set at a speed between medium and fast. Firstly, the clear height distribution pattern (face height and ground level) suggests that the placement of a fan might be dictated by space and equipment constraints as was evident by ∼40% of participants responding that their selected fan position was not in the place they wanted it to be. However, having the fan directed towards the face is considered an optimal strategy for thermal comfort as this area has the highest thermos-sensitivity within the body (33). Participants estimated that fan blade diameter was between 20 and 30 cms. The upper range rotational velocity for an indoor pedestal fan blade is typically reported between 1,300–1,400 rpm according to manufacturer guidelines, suggesting that air velocity would approximate ∼6.5 m/s which is in line with published data on the required air flow needed to optimize exercise in the heat (34). Interestingly only one respondent in the entire sample directly altered humidity, reporting use of a dehumidifier, which alters the evaporation efficiency blunting mechanisms for heat loss. In enclosed indoor spaces it is likely air humidity increases, therefore, further investigation is warranted.

Whilst control of ambient conditions was well reported, strategies to modulate body temperature were less well understood with only ∼30% of participants indicating they had attempted to directly change body temperature. It is well established that body cooling prior to exercise can extend exercise performance (35). Those who did consider lowering body temperature used a mixed approach of cold air exposure and cold ice/fluid ingestion. Whilst raising body temperature was achieved by wearing extra clothing. Interestingly, only 16% of respondents have used indoor training to prepare for hot climates, which would certainly warrant further investigation, especially given recent findings that heat training could also positively impact haemoglobin mass when completing a short exercise program either in a hot space or by wearing a heat suit (36).

Dehydration exacerbates the rise in core body temperature and thus the development of fatigue (37). 98% of participants reported consuming a drink during indoor cycling, with mean ingestion of ∼0.74 ml/h. The average person would expect to lose ∼0.4–1.8 L/h in sweat during exercise, although this is highly variable between individuals (38). Guidelines for fluid ingestion are typically∼0.4–0.8 L/h (38), meaning that in our sample participants fluid ingestion is generally adequate to offset dehydration.

### 4.3 Training habits

Despite the popularity of people engaging in home-based indoor cycling (1) there is little data examining how people engage in this form of exercise. Here, we report that the average training session duration was ∼72 min, with participants engaging in indoor cycling between 1 and 2 times per week. However, a significant difference in amount of weekly indoor cycling hours was evident between summer (∼2 h 54 min) and winter (∼6 h 12 min) months. There was also a difference in the time of day sessions were completed, with a shift towards the evening in the winter months. These practices coincide with the primary reasons that people engage in indoor cycling which was reported as: weather (88%) and a lack of daylight (56%). It should be noted that a large proportion of the study sample live in temperate climates and are likely more affected by seasonal fluctuations in weather and daylight, thus, reasons for indoor cycling may differ at different latitudes. Additionally, time-efficiency (81.9%) and general fitness (70.9%) were indicated as primary reasons to engage in indoor cycling. Indeed, >80% of respondents indicated that sessions were conducted in the morning or evening, which may relate to planning sessions around work commitments. Together with the finding that participants mainly engage in structured work-outs once or twice a week (∼60%.), it would highlight the notion that indoor cycling can be used to incorporate high-intensity interval training (HIIT) as a time-efficient way to exercise (39). Although causality cannot be established using the current study design, aerobic fitness (FTP) was positively correlated with the frequency of engaging in both structured work-outs and racing events, whilst negatively correlated with engagement in unstructured sessions. In combination with the significantly higher reported RPEs during racing and work-outs, this indicates that exercise intensity is an important driver for improving fitness if matched for work (kJ) (40). However, whilst there have been several high-profile professional online competitive events (2) which could stimulate greater engagement in competitive racing online, only ∼20% of the sample population had entered competitive online races. 30% of people indicated that they have never engaged in racing events. This could be for several reasons, such as the mixed-reality software not offering competition, the very-high perceived effort of the session (8.6/10), but also due to possible concerns of cheating using so-called “weight-doping”, the practice of decreasing body weight settings in the software to increase relative power output (w/kg) (11, 41). One limitation of these findings is that data was all self-reported without cross-validation for objective data. Extending time to complete the questionnaire by requesting participants to access their exercise diary or training application software to self-validate answers could have reduced completion rates, whilst providing access to researchers for validation would remove participant anonymity. Future studies should aim to validate the accuracy of self-reported exercise activities for home-based indoor cycling.

### 4.4 Nutritional habits

Carbohydrate intake is important in endurance performance and recommendations for carbohydrate fueling before, during and after endurance exercise often reflect exercise duration and intensity (42, 43). We asked participants to report nutritional strategies used for session type (race, work-out, unstructured ride) and duration (< 1 h >) to better understand their nutritional habits. The proportion of participants who did not plan carbohydrate intake prior to their exercise session was lower in longer-duration (19.6–20.6%) than short-duration (36.4–58.4%) exercise (Table 2). Participants who did report carbohydrate consumption during exercise, did so at an average rate of ∼35 g/hour, which is in line with recommendations (42) and has been previously reported for indoor cycling (13). Irrespective of the duration of the session, deliberate planning of carbohydrate intake around the exercise session was higher for racing events (24.6–65.3%) and structured work-outs (20.3% - 61.6%), than for unstructured sessions (15.1% - 56.8%%), aligned with the greater demands of these sessions. Therefore, in the sample studied it does appear that participants are aware of tailoring their carbohydrate intake based on the duration and intensity of exercise performed. However, it is relevant to note that still 1 in 5 participants do not have a plan for carbohydrate supplementation during the most demanding sessions (Table 2).

The consumption of protein is generally considered for post-exercise recovery however emerging research suggests that protein supplementation prior to and during exercise may also influence performance and training adaptation (44, 45). However, it is unsurprising that deliberate and intentional intake of protein largely after exercise was reported by ∼50% of the participants. Greater numbers were reported in longer duration exercise compared to shorter duration exercise and in racing and structured work-out sessions compared to unstructured sessions. Of those who consume protein post-exercise, an average intake of ∼0.35 g of protein per kg body weight (e.g.,∼28 g for an 80 kg individual) was reported which is in line with general recommendations on protein supplementation to promote muscle remodeling in endurance exercise (46). Protein supplementation prior or during exercise was low < 10%. It was noted that a number of open-text comments referred to fasted exercise when training indoors. Recently, it has been suggested that protein intake before exercise results in similar metabolic effects as fasted exercise (47), therefore this could be an area of attention for indoor cyclists doing fasted training.

Based on the IOC consensus statement for dietary supplements in high performance athletes (48) and consensus guidelines on the use of menthol supplements during exercise in warm environments (49, 50), participants were asked if they had ever used supplements during indoor cycling sessions. Caffeine was reported to be used by ∼63% of participants, which is in line with previous questionnaires (51, 52), although these questionnaires didn't address indoor cycling specifically. We did not however distinguish whether the source of caffeine was supplemental or in the form of a drink (fizzy drink/coffee/tea) therefore we cannot validate whether the dose used is in line with current recommendations. Across the other supplements we examined, >85% of the participants stated they had never used these supplements during indoor cycling, which supports previous evidence (51).

### 4.5 Future directions

The findings from this research have revealed a broad range of data that may provide future directions for research, insight for individuals exercising indoors, or the development of evidence-based indoor training guidelines. Firstly, more research into validating the accuracy of different models of smart trainers would be required to support consistency in equipment and data gathering. Secondly, in the area of environmental control, despite the common use of fans and preference for placement directly in front, more variability existed in the height and distance, likely dictated by space constraints, thus clearer evidence-based guidance would be beneficial. Additionally, for people who consider using indoor cycling to promote heat adaptation, further guidelines for safety need to be developed. Reinforcement of optimal nutritional strategies for indoor cycling would be also beneficial to this emerging exercise community. The popularity of home-based indoor cycling and findings presented here should facilitate the development and optimization of future research utilizing the indoor-cycling community in home-based studies to maximize sample sizes as has recently been completed during COVID-19 (53–55). For example, our findings provide insightful data to help facilitate optimization of adherence to proposed protocols. As engagement with indoor cycling is greater during the winter months due to lack of daylight and weather events, researchers could specifically plan to run their study during this time window to improve adherence. Additionally, with findings suggesting that morning and evening are the most popular times to engage in indoor cycling, this could support the experimental designs looking into research investigating circadian rhythms and best time of day to exercise (56, 57), potentially in relation to nutrition (58). Lastly, the impact psychological elements linked to engagement with indoor cycling performance, for example motivation and mental fatigue (59), could be another area for future research.

## 5 Conclusion

Home-based indoor cycling using mixed-reality software has grown in popularity with ongoing developments in gamification. Using a cross-sectional questionnaire in a large population sample, we have (i) highlighted areas of consistent implementation of habits in line with exercise science, including use of fans to aid in temperature and fluid consumption, (ii) highlighted areas of inconsistency such as planning and nutrition, and (iii) identified opportunities and provided suggestions for further research.

## Data Availability

The raw data supporting the conclusions of this article will be made available by the authors, without undue reservation.
