# Supplementary material for: Training, environmental and nutritional practices in indoor cycling: an explorative cross-sectional questionnaire analysis
Source: Front Sports Act Living. 2024 Oct 11;6:1433368. doi: 10.3389/fspor.2024.1433368 (PMC11502339; doi:10.3389/fspor.2024.1433368)
Supplement: Supplementary file 1 [file Table1.docx]

Supplementary Material

# Questionnaire

Indoor Cycling Project

Survey Flow

Standard: About the Survey (1 Question)

Block: Background Information (13 Questions)

Standard: PART ONE- Equipment set-up (13 Questions)

Standard: Environment (19 Questions)

Standard: Part Two - Training (15 Questions)

Standard: Nutrition (49 Questions)

Start of Block: About the Survey

Q1 **What is the purpose of the study?** This study aims to gather information about common practices of people who complete cycling sessions at home, this could include using a virtual cycling platform. The questionnaire is split in two parts. Part One will ask you about your set up and Part Two will ask about training sessions and nutritional strategies.    **Why have I been invited?** You have been invited to participate in the study as you are 18+ years old and participate in indoor cycling (cycling does not have to be your primary sport).    **What would be involved for me?** Simply access the link provided via computer, laptop, or smart phone, and fill out the online questionnaire. Part One of the questionnaire will take about 5 - 10 minutes to complete which will include demographical questions and questions on your indoor cycling habits, set up and environment. Following completion of the primary questionnaire, we would be grateful if you could complete Part Two, which will take an extra 5 - 10 minutes, this will ask you about your training sessions and nutrition.   
 As the participant you will have up to 14 days to access the questionnaire and complete the form before data is captured and protected in a safe document file.   **What are the possible benefits of taking part?** The benefits of participating in this study will include a greater awareness of how you train indoors using a virtual cycling platform, it will stimulate reflection on your indoor cycling environment, and what you eat and drink whilst cycling.   **What are the possible disadvantages and risks of participation?** There are no risks associated with this online questionnaire and all questions have been reviewed and approved by the research team. If any of the questions were to make you feel uncomfortable then you should stop what you are doing and consider withdrawing from the study.   **Do I have to take part?** Your participation in this study is entirely voluntary. If you do decide to participate, you have the right to withdraw from the study at any point in time.    **What do I need to do if I wish to take part?** Please read this Information Sheet and ask any questions that you may have about the process, by emailing the project team leader (email below). If you are happy to be involved then please read and provide informed consent by selecting the box below.   **Will my participation in the study be kept confidential?** You are not requested to supply your name at any point, therefore at no stage will your name be revealed in any part of the study and no reference will be made which could link you to the study. All information you provide will be handled in strict confidence, and will be seen only by the research team.   **What if I have any concerns or queries?** For issues relating to the project, please contact the project leader: This study was approved by Local University's Ethics Committee [Ref: 26899/2022].       Thank you for taking the time to read this information.    If you are happy to proceed with this study please consent by ticking the box below.

- I consent to participate in the study (1)
- I do not consent, I do not wish to participate (2)

Skip To: End of Survey If What is the purpose of the study? This study aims to gather information about common practices of... = I do not consent, I do not wish to participate

End of Block: About the Survey

Start of Block: Background Information

Q2 Please state your gender

- Male (1)
- Female (2)
- Other (3)
- Prefer not to say (4)

Display This Question:

If Please state your gender = Other

Q3 If other, please state

________________________________________________________________

Q4 What is your age?

|  | 18 | 26 | 34 | 43 | 51 | 59 | 67 | 75 | 84 | 92 | 100 |
| --- | --- | --- | --- | --- | --- | --- | --- | --- | --- | --- | --- |

| Years () | 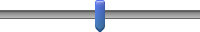 |
| --- | --- |

Q5 What is your body mass/weight in kg
(1 stone = 6.35 kg)?

|  | 30 | 42 | 54 | 66 | 78 | 90 | 102 | 114 | 126 | 138 | 150 |
| --- | --- | --- | --- | --- | --- | --- | --- | --- | --- | --- | --- |

| In Kg () | 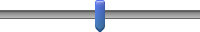 |
| --- | --- |

Q6 What best describes your ethnicity?

- White (1)
- Mixed/Multiple Ethnic backgrounds (21)
- Asian (22)
- Black/African/Caribbean (23)
- Other ethnic group (24)
- Prefer not to say (25)

Q7 What country do you live in?

▼ Afghanistan (1) ... Zimbabwe (198)

Q8 How would you describe your current cycling status?

- Recreational (8)
- Amateur non-competitor (31)
- Amateur competitor (12)
- National level competitor (9)
- International level competitor (10)
- Professional (11)

Q9 How many *hours* per week do you exercise over a year on average?

|  | 1 | 7 | 13 | 18 | 24 | 30 |
| --- | --- | --- | --- | --- | --- | --- |

| Hours () | 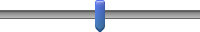 |
| --- | --- |

Q10 How many *hours* per week do you cycle over a year on average?

|  | 1 | 7 | 13 | 18 | 24 | 30 |
| --- | --- | --- | --- | --- | --- | --- |

| Hours () | 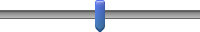 |
| --- | --- |

Q11 How many *hours* per week do you cycle **indoors** over a year on average?

|  | 1 | 7 | 13 | 18 | 24 | 30 |
| --- | --- | --- | --- | --- | --- | --- |

| Hours () | 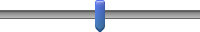 |
| --- | --- |

Q12 Do you know what your Functional Threshold Power (FTP) is in Watts?

FTP can be obtained in several ways. Most popular examples include:
1. 95% of 20-minute maximal power output test
2. Predicted FTP from training software

Please use your latest test result. If you do not know or can't remember, please leave blank.

|  | 100 | 140 | 180 | 220 | 260 | 300 | 340 | 380 | 420 | 460 | 500 |
| --- | --- | --- | --- | --- | --- | --- | --- | --- | --- | --- | --- |

| In Watts () | 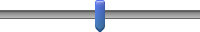 |
| --- | --- |

Q13 Why do you engage in indoor cycling sessions? Select all reasons that apply.

- Injury recovery/Rehabilitation (2)
- Social interaction (3)
- Technique training (4)
- Time efficiency (5)
- Concerns about road safety (6)
- Performance testing (7)
- Weather (8)
- No interruption/consistent work-out (9)
- Lack of daylight (10)
- General fitness (12)
- Competion/Racing (15)
- Other (16)

Display This Question:

If Why do you engage in indoor cycling sessions? Select all reasons that apply. = Other

Q14 What other reasons do you have why you engage in indoor cycling

________________________________________________________________

End of Block: Background Information

Start of Block: PART ONE- Equipment set-up

Q15 The following questions will ask you about the equipment and software that you use for your indoor cycling sessions. Please try to answer these to the best of your abilities.

Q16 What type of indoor cycling trainer do you use? Use the image below for clarification: (1) direct drive, (2) wheel-on, (3) roller, (4) smart bike.

- 1. Direct drive smart trainer (1)
- 2. Wheel-on turbo trainer (2)
- 3. Rollers (3)
- 4. Smart bike (4)

Q17 What virtual cycling platform/software do you use? If you use multiple, select the one that you use most often.

▼ BKool (1) ... I don't use a virtual cycling platform (12)

Display This Question:

If What virtual cycling platform/software do you use? If you use multiple, select the one that you u... = Other

Q18 What other type of virtual cycling platform do you use?

________________________________________________________________

Q19 What interface/device do you primarily use to display your virtual cycling platform when exercising?

- Laptop (1)
- TV (2)
- Tablet (3)
- Mobile phone (4)
- Projector (5)
- Other (6)
- I don't use an interface/device to display the virtual cycling platform (7)

Skip To: Q25 If What interface/device do you primarily use to display your virtual cycling platform when exercising? = I don't use an interface/device to display the virtual cycling platform

Display This Question:

If What interface/device do you primarily use to display your virtual cycling platform when exercising? = Other

Q20 What other interface/device do you use to display your virtual cycling platform?

________________________________________________________________

Q21 What position best describes the height and position at which your interface/device is located? Please select the number that best describes the position in the image below.


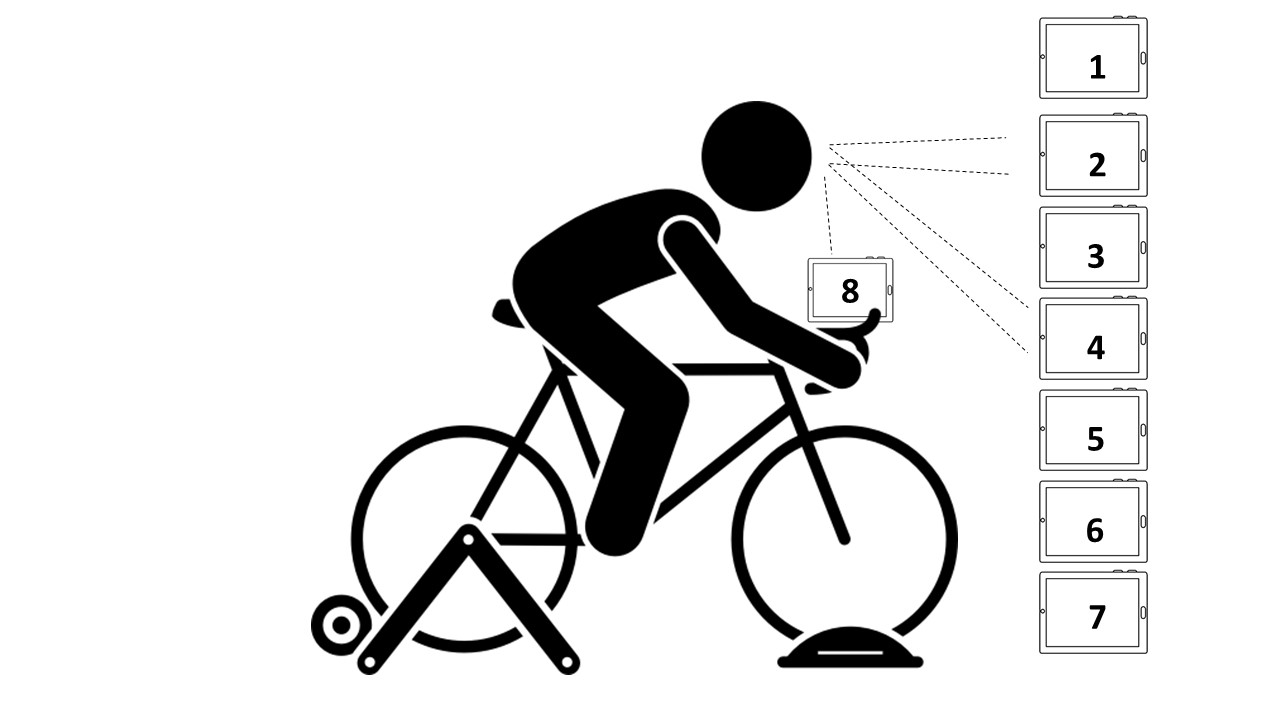


- 1 (1)
- 2 (2)
- 3 (3)
- 4 (4)
- 5 (5)
- 6 (6)
- 7 (7)
- 8 (8)
- The height of my device is not reflected in the image (9)

Skip To: Q24 If What position best describes the height and position at which your interface/device is located? P... = The height of my device is not reflected in the image

Q22 What is the primary reason for the height and position of your interface/device?

- Space/room constraints (1)
- Injury/rehabilitation purpose (2)
- Neck/back strengthening purpose (4)
- Eye-sight purpose (5)
- Performance optimisation (6)
- Other (8)

Display This Question:

If What is the primary reason for the height and position of your interface/device? = Other

Q23 What other reason is there for the purpose of the interface/device positioning?

________________________________________________________________

Q24 Where do you place your device for viewing the virtual content? Please select the number that best describes the position in the picture below.


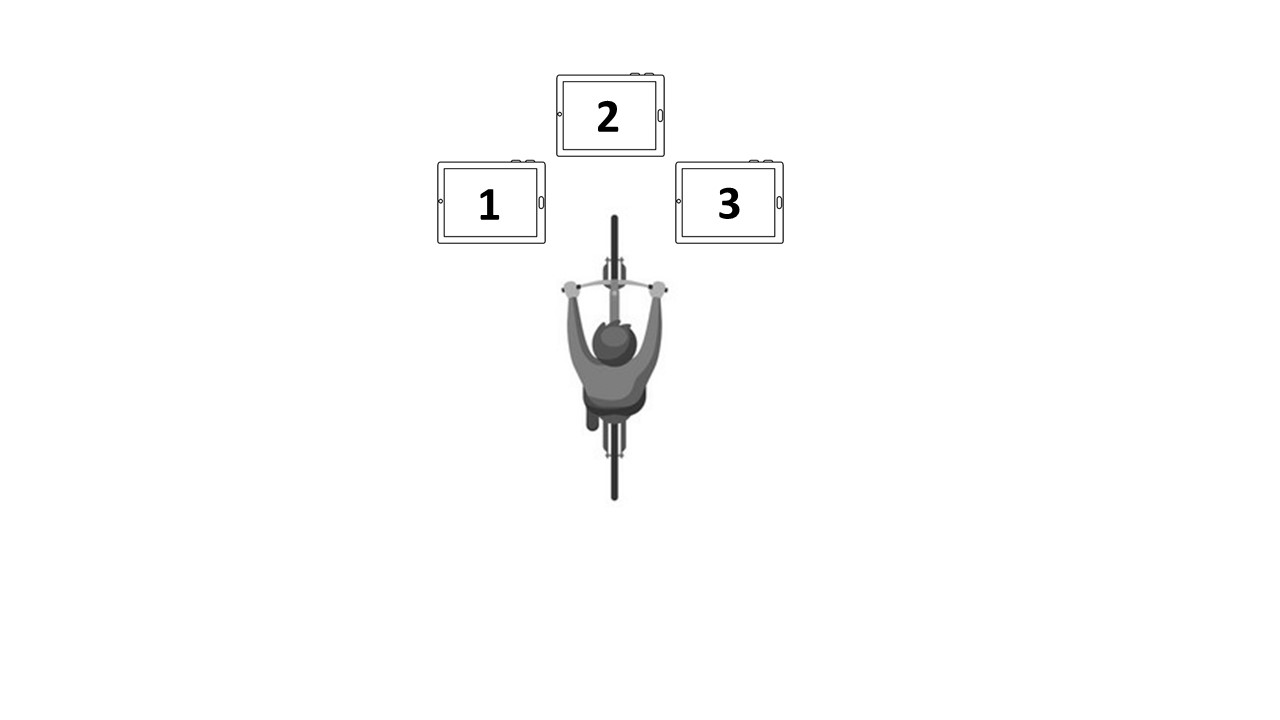


- 1 (1)
- 2 (2)
- 3 (3)
- The position of my interface is not reflected in the image (4)

Q25 Do you use a rocker plate to mimic outdoor cycling? Example image below.

- Yes (1)
- No (2)

Q26 Do you use any equipment or software to change the gradient on your bike to mimic climbing or descending?

- Yes (1)
- No (2)

Q27 Are there any other practices relating to your equipment set up during your indoor cycling sessions that you **deliberately** and **typically** engage in that have not been covered by the questionnaire? *Equipment for temperature control and nutritional interventions are covered in the next set of questions.

Please use the text entry to describe these practices. If this is not the case, you can move to the next question and leave this section blank.

________________________________________________________________

End of Block: PART ONE- Equipment set-up

Start of Block: Environment

Q28 The next set of questions will ask you about the environment that you conduct your indoor cycling sessions in and attempts to control the environment. Please try to answer these questions to the best of your ability.

Q29 Do you attempt to control room temperature/air movement in the space that you exercise in? Select all that apply

- Yes, I attempt to control the room temperature/air movement during the summer (1)
- Yes, I attempt to control the room temperature/air movement during the winter (2)
- No, I do not attempt to control the room temperature/air movement (3)

Skip To: Q42 If Do you attempt to control room temperature/air movement in the space that you exercise in? Select... = No, I do not attempt to control the room temperature/air movement

Display This Question:

If Do you attempt to control room temperature/air movement in the space that you exercise in? Select... = Yes, I attempt to control the room temperature/air movement during the winter

Q30 In *winter*, how do you attempt to control room temperature/air movement of the room that you exercise in? Select all that apply

- Open windows (1)
- Air conditioner (3)
- Central heating (5)
- Fan (11)
- Move to a warmer room (12)
- Move to a cooler room (13)
- Other (14)

Display This Question:

If In winter, how do you attempt to control room temperature/air movement of the room that you exerc... = Other

Q31 What other ways do you control room temperature/air movement of the room during winter?

________________________________________________________________

Display This Question:

If Do you attempt to control room temperature/air movement in the space that you exercise in? Select... = Yes, I attempt to control the room temperature/air movement during the summer

Q32 In *summer*, how do you attempt to control room temperature/air movement of the space that you exercise in? Select all that apply

- Open windows (1)
- Air conditioner (3)
- Fan (5)
- Move to a warmer room (8)
- Move to a cooler room (9)
- Other (10)

Display This Question:

If In summer, how do you attempt to control room temperature/air movement of the space that you exer... = Other

Q33 What other ways do you control room temperature/air movement of the space during summer?

________________________________________________________________

Q34 If you do use a fan, how many fans do you use? If you don't use a fan, please select 0.

|  | 0 | 1 | 2 | 3 | 4 | 5 | 6 |
| --- | --- | --- | --- | --- | --- | --- | --- |

| Number of fans () | 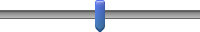 |
| --- | --- |

Skip To: Q42 If If you do use a fan, how many fans do you use? If you don't use a fan, please select 0. [ Number of fans ] <=

Q35 Please estimate the diameter (length end to end) of the blades/rotor. If you are not sure, please leave this answer blank.

|  | 0 | 10 | 20 | 30 | 40 | 50 | 60 |
| --- | --- | --- | --- | --- | --- | --- | --- |

| Blade diameter (in cms) () | 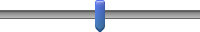 |
| --- | --- |

Q36 Please estimate the distance (in metres) between the handlebar and the fan?

- 0.5 - 1 m (1)
- 1 - 1.5 m (2)
- 1.5 - 2 m (3)
- Greater than 2 m (4)

Q37 From the image below, select the number that best reflects the most frequently used position of your fan - select all that apply if you use multiple fans.


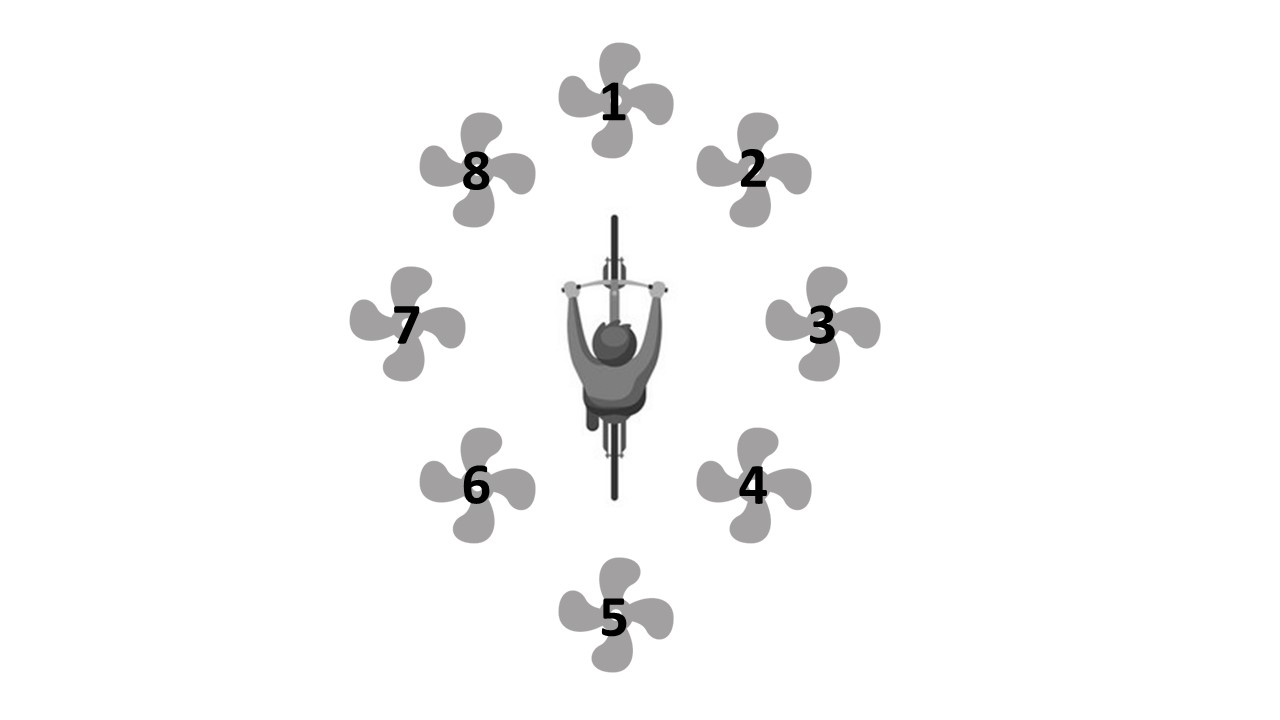


- Position 1 (1)
- Position 2 (2)
- Position 3 (3)
- Position 4 (4)
- Position 5 (5)
- Position 6 (6)
- Position 7 (7)
- Position 8 (8)

Q38 From the image below, select the number that best reflects the most frequent height you have positioned your fan - select all that apply if you use multiple fans.


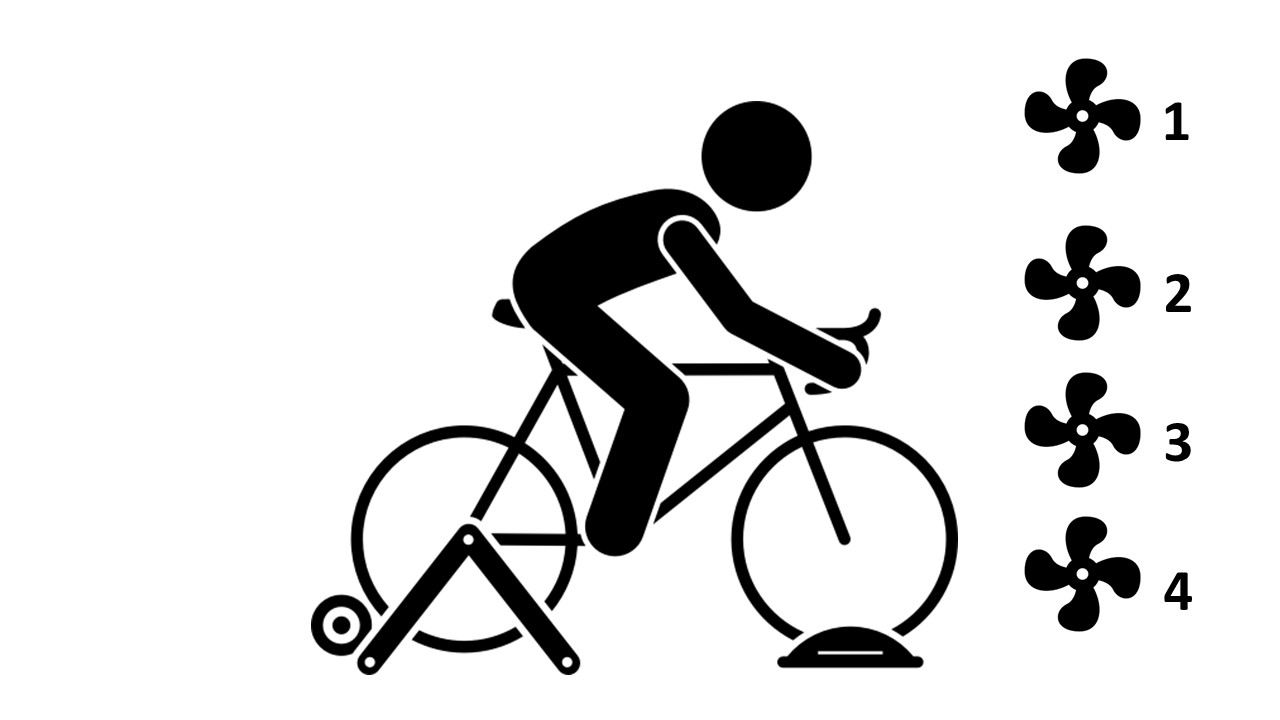


- Position 1 (1)
- Position 2 (2)
- Position 3 (3)
- Position 4 (4)

Q39 Is the position and height of your fan in the place that you want it to be?

- Yes, I think that the placement of the fan(s) is optimal (1)
- No, there are space constraints (2)
- No, there are equipment constraints (4)
- Other (5)

Display This Question:

If Is the position and height of your fan in the place that you want it to be? = Other

Q40 What is the other reason why you consider the placement of the fan not to be optimal?

________________________________________________________________

Q41 What fan speed setting do you use when cycling indoors? (Fans might have different speed settings (e.g. slow / moderate / fast speeds)).

- Lowest speed setting (1)
- Moderate speed setting (2)
- Highest speed setting (3)

Q42 The previous questions asked you about controlling room temperature in the space you exercise in. Have you ever attempted to deliberately control your **body temperature** *prior* or *during* indoor cycling exercise by any of the following means? (select any that apply)

- Lower body temperature: cooling vest (1)
- Lower body temperature: exposure to cold water/ice bath (2)
- Lower body temperature: exposure to cold air (3)
- Lower body temperature: consumption of chilled fluids/ice (4)
- Raise body temperature: extra clothing (6)
- Raise body temperature: exposure to warm water/hot bath (7)
- Raise body temperature: consumption of hot fluids (8)
- Raise body temperature: exposure to warm air (9)
- I have never attempted to lower body temperature (11)
- I have never attempted to raise body temperature (12)

Q43 Have you ever used indoor cycling exercise to improve heat tolerance in preparation for an event in a hot climate?

- Yes (1)
- No (7)

Q44 What clothing do you wear during the majority of your indoor cycling sessions (excluding underwear eg. sports bra)?

- Shorts only (1)
- Shorts and base layer (2)
- Shorts and base layer and jersey (short sleeve) (3)
- Shorts and base layer and jersey (long sleeve) (4)

Q45 Are there any other environmental practices around indoor cycling sessions that you **deliberately** and **typically** engage in that have not been covered by the questionnaire?

Please use the text entry to describe this practice. If this is not the case, you can move to the next question and leave this section blank.

________________________________________________________________

Q46 You now have reached the end of the Part One, thank you! You can now decide whether you would be willing to continue with a series of questions asking you about your training and nutritional practices during indoor cycling. It will take approximately 5-10 minutes to complete these questions. If you do not want to complete these questions, you can go to the end of the survey.

Do you wish to continue completing questions about your training and nutritional practices during indoor cycling?

- Yes, I want to continue with these questions (1)
- No, I want to go to the end of the survey (2)

Skip To: End of Survey If You now have reached the end of the Part One, thank you! You can now decide whether you would be... = No, I want to go to the end of the survey

End of Block: Environment

Start of Block: Part Two - Training

Q47 The following questions will ask you about your **indoor cycling** training. Please answer the questions to the best of your abilities.

Q48 On average, how long are your indoor cycling sessions (in minutes)?

|  | 0 | 30 | 60 | 90 | 120 | 150 | 180 | 210 | 240 | 270 | 300 |
| --- | --- | --- | --- | --- | --- | --- | --- | --- | --- | --- | --- |

| Duration of average indoor cycling session (in minutes) () | 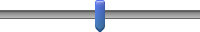 |
| --- | --- |

Q49 How often do you complete an indoor cycling session that is 1 hour or less?

- Never (1)
- Less than once a month (2)
- Once a month (3)
- Twice a month (4)
- Once a week (5)
- Twice or more a week (6)

Q50 How often do you complete an indoor cycling session that is longer than 1 hour?

- Never (1)
- Less than once a month (2)
- Once a month (3)
- Twice a month (4)
- Once a week (5)
- Twice or more a week (6)

Q51 What is the longest indoor cycling session you have ever completed (please round up to the nearest hour)?

|  | 0 | 2 | 4 | 6 | 8 | 10 | 12 | 14 | 16 | 18 | 20 | 22 | 24 |
| --- | --- | --- | --- | --- | --- | --- | --- | --- | --- | --- | --- | --- | --- |

| Duration of longest indoor cycling session (in hours) () | 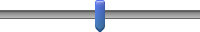 |
| --- | --- |

Q52 On average, how many **hours** per week during the colder winter months do you cycle indoors?

|  | 0 | 1 | 2 | 3 | 4 | 5 | 6 | 7 | 8 | 9 | 10 | 11 | 12 | 13 | 14 | 15 | 16 | 17 | 18 | 19 | 20 |
| --- | --- | --- | --- | --- | --- | --- | --- | --- | --- | --- | --- | --- | --- | --- | --- | --- | --- | --- | --- | --- | --- |

| Hours per week () | 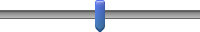 |
| --- | --- |

Skip To: Q54 If On average, how many hours per week during the colder winter months do you cycle indoors? [ Hours per week ] <=

Q53 At what time of day do you complete *most* of your indoor cycling sessions during the winter months?

- In the morning (earlier than 11:00/11AM) (1)
- In the afternoon (between 11:00/11AM and 16:00/4PM) (2)
- In the evening (after 16:00/4PM) (3)

Q54 On average, how many hours per week during the warmer summer months do you cycle indoors?

|  | 0 | 1 | 2 | 3 | 4 | 5 | 6 | 7 | 8 | 9 | 10 | 11 | 12 | 13 | 14 | 15 | 16 | 17 | 18 | 19 | 20 |
| --- | --- | --- | --- | --- | --- | --- | --- | --- | --- | --- | --- | --- | --- | --- | --- | --- | --- | --- | --- | --- | --- |

| Hours per week () | 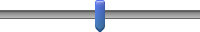 |
| --- | --- |

Skip To: Q56 If On average, how many hours per week during the warmer summer months do you cycle indoors? [ Hours per week ] <=

Q55 At what time of day do you complete *most* of your indoor cycling sessions during the summer months?

- In the morning (earlier than 11:00/11AM) (1)
- In the afternoon (between 11:00/11AM and 16:00/4PM) (2)
- In the evening (after 16:00/4PM) (3)

Q56 The next questions will ask you about the types of indoor cycling sessions you complete whether using a virtual cycling software, or not. Therefore, we define the following types of indoor cycling sessions as:

 1. **A virtual racing event**: A virtual racing events is defined as any event with a competitive element/competing against other cyclists or avatars.
 2. **A structured work-out session**: A structured work-out session is defined as a pre-specified or pre-built work-out (e.g. intervals, or sprints, etc...)
 3. **An unstructured cycling session**: An unstructured ride is defined as anything other than a racing event or a structured workout session (e.g. social ride/group ride, recovery spin).

Q57 How often do you enter virtual racing events? A virtual racing events is defined as any event with a competitive element/competing against other cyclists or avatars.

- Never (3)
- Less than once a month (4)
- Once a month (5)
- Twice a month (6)
- Once a week (7)
- Twice or more a week (8)
- My virtual cycling platform does not offer racing events (11)

Q58 How often do you perform structured work-out sessions? A structured work-out session is defined as a pre-specified or pre-built work-out (e.g. intervals, or sprints, etc...)

- Never (1)
- Less than once a month (2)
- Once a month (3)
- Twice a month (4)
- Once a week (5)
- Twice or more a week (6)
- My virtual cycling platform does not offer structured work-out sessions (7)

Q59 How often do you cycle unstructured? An unstructured ride is defined as anything other than a racing event or a structured workout session (e.g. social ride/group ride, recovery spin).

- Never (1)
- Less than once a month (2)
- Once a month (3)
- Twice a month (4)
- Once a week (5)
- Twice or more a week (6)
- My virtual cycling platform does not offer unstructured cycling options (7)

Q60 On a scale of 1 to 10 (1 being the minimum effort and 10 being maximum effort), what number would you use to describe the perceived effort you experience on average during the different types of indoor cycling sessions?

If you don't complete one of these sessions, please select not applicable.

|  | Not Applicable |
| --- | --- |

|  | 1 | 2 | 3 | 4 | 5 | 6 | 7 | 8 | 9 | 10 |
| --- | --- | --- | --- | --- | --- | --- | --- | --- | --- | --- |

| VIRTUAL RACING () | 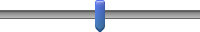 |
| --- | --- |
| STRUCTURED WORK-OUT () | 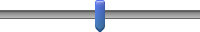 |
| UNSTRUCTURED () | 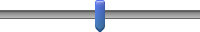 |

Q61 Are there any other indoor cycling session practices around training that you **deliberately** and **typically** engage in that have not been covered by the questionnaire?

Please use the text entry to describe this practice. If this is not the case, you can move to the next question and leave this section blank.

________________________________________________________________

End of Block: Part Two - Training

Start of Block: Nutrition

Q62 In this final section, the following questions will ask you about how you manage your nutrition during your indoor cycling sessions. Please answer the questions to the best of your abilities.

Q63 Do you consume fluids/drinks during your indoor cycling session?

- Yes (1)
- No (2)

Skip To: Q67 If Do you consume fluids/drinks during your indoor cycling session? = No

Q64 To the best of your ability, please estimate how much fluid you generally drink ***per hour*** during an indoor cycling session. If you are unsure, please use the image below that can help with estimating the fluid intake based on the size of a bidon/bottle and if necessary multiply by the amount of bottles you consume per hour. (1000 ml in 1 L).

|  | 0 | 0 | 0 | 1 | 1 | 1 | 1 | 1 | 2 |
| --- | --- | --- | --- | --- | --- | --- | --- | --- | --- |

| Volume per hour (in litres) () | 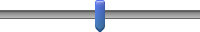 |
| --- | --- |

Q65 Does your drink typically contain or do you deliberately add any of the following? Please select all that apply

- Carbohydrates/sugar (1)
- Electrolytes/salt (2)
- Other (specify next) (3)
- No, I just drink water (4)

Display This Question:

If Does your drink typically contain or do you deliberately add any of the following? Please select... = Other (specify next)

Q66 What else do you typically add to your drink, or that your drink contains?

________________________________________________________________

Q67 In the next questions, information relating to nutritional practices is gathered based on the duration and type of your indoor cycling sessions. We define the following sessions based on duration as:

 1. **Short-duration**: indoor cycling sessions lasting 1 hour or less
 2. **Long-duration**: indoor cycling sessions lasting for longer than 1 hour. 

The type of session has been defined before: racing event, structured work-out and unstructured session.

Most people will consume food in close proximity to an exercise session as part of their normal lifestyle (e.g. breakfast, lunch, dinner). When answering the next set of questions, only select "yes" if you consume food/drinks with the intention to support your exercise session and recovery from the session. If you do not intentionally consume food/drinks around the exercise with the goal to support/recover from your exercise session, please select "I don't plan/I don't have a strategy for this event".

Q68 The following statements will ask you about your practices around ***carbohydrate*intake** for the different indoor cycling sessions. Practices will be separated based on type (racing event, structured work-out, unstructured session) and duration (short-duration < 1h, long-duration >1h)

Please indicate whether you *intentionally* consume carbohydrates to support your exercise session. Select all time points that apply.

|  | Before (1) | During (2) | After (3) | I don't plan/I don't have a strategy for this event (4) | I have never engaged in such a session (5) |
| --- | --- | --- | --- | --- | --- |
| For a virtual racing event shorter than 1h, I intentionally consume carbohydrates: (1) |  |  |  |  |  |
| For a structured work-out session shorter than 1h, I intentionally consume carbohydrates: (2) |  |  |  |  |  |
| For an unstructured session shorter than 1h, I intentionally consume carbohydrates: (3) |  |  |  |  |  |
| For a virtual racing event longer than 1h, I intentionally consume carbohydrates: (5) |  |  |  |  |  |
| For a structured work-out session longer than 1h, I intentionally consume carbohydrates: (6) |  |  |  |  |  |
| For an unstructured session longer than 1h, I intentionally consume carbohydrates: (7) |  |  |  |  |  |

Display This Question:

If The following statements will ask you about your practices around carbohydrate intake for the dif... = For a virtual racing event shorter than 1h, I intentionally consume carbohydrates: [ Before ]

Q69 You indicated that you intentionally consume carbohydrates *before* a short-duration (<1h) racing event. Please estimate how much carbohydrate you usually consume before a short-duration session (in grams).

As reference, carbohydrate content is: medium banana ~30 g; Lucozade sport drink ~30 g; Mars bar ~38 g; standard energy gel ~20 g.

- 20 grams (7)
- 30 grams (1)
- 60 grams (2)
- 90 grams (3)
- > 90 grams (4)
- I don't know (6)

Display This Question:

If The following statements will ask you about your practices around carbohydrate intake for the dif... = For a structured work-out session shorter than 1h, I intentionally consume carbohydrates: [ Before ]

Q70 You indicated that you intentionally consume carbohydrates *before* a short-duration (<1h) structured work-out. Please estimate how much carbohydrate you usually consume before a short-duration session (in grams).

As reference, carbohydrate content is: medium banana ~30 g; Lucozade sport drink ~30 g; Mars bar ~38 g; standard energy gel ~20 g.

- 20 grams (7)
- 30 grams (1)
- 60 grams (2)
- 90 grams (3)
- > 90 grams (4)
- I don't know (6)

Display This Question:

If The following statements will ask you about your practices around carbohydrate intake for the dif... = For an unstructured session shorter than 1h, I intentionally consume carbohydrates: [ Before ]

Q71 You indicated that you intentionally consume carbohydrates *before* a short-duration (<1h) unstructured session. Please estimate how much carbohydrate you usually consume before a short-duration session (in grams).

As reference, carbohydrate content is: medium banana ~30 g; Lucozade sport drink ~30 g; Mars bar ~38 g; standard energy gel ~20 g.

- 20 grams (7)
- 30 grams (1)
- 60 grams (2)
- 90 grams (3)
- > 90 grams (4)
- I don't know (6)

Display This Question:

If The following statements will ask you about your practices around carbohydrate intake for the dif... = For a virtual racing event shorter than 1h, I intentionally consume carbohydrates: [ During ]

Q72 You indicated that you intentionally consume carbohydrates *during* a short-duration (<1h) racing event. Please estimate how much carbohydrate you usually consume during a short-duration session (in grams/hour).

As reference, carbohydrate content is: medium banana ~30 g; Lucozade sport drink ~30 g; Mars bar ~38 g; standard energy gel ~20 g.

- 20 grams per hour (7)
- 30 grams per hour (1)
- 60 grams per hour (2)
- 90 grams per hour (3)
- > 90 grams per hour (4)
- I don't know (6)

Display This Question:

If The following statements will ask you about your practices around carbohydrate intake for the dif... = For a structured work-out session shorter than 1h, I intentionally consume carbohydrates: [ During ]

Q73 You indicated that you intentionally consume carbohydrates *during* a short-duration (< 1h) structured work-out. Please estimate how much carbohydrate you usually consume during a short-duration session (in grams/hour).

As reference, carbohydrate content is: medium banana ~30 g; Lucozade sport drink ~30 g; Mars bar ~38 g; standard energy gel ~20 g.

- 20 grams per hour (7)
- 30 grams per hour (1)
- 60 grams per hour (2)
- 90 grams per hour (3)
- > 90 grams per hour (4)
- I don't know (6)

Display This Question:

If The following statements will ask you about your practices around carbohydrate intake for the dif... = For an unstructured session shorter than 1h, I intentionally consume carbohydrates: [ During ]

Q74 You indicated that you intentionally consume carbohydrates *during* a short-duration (<1h) unstructured session. Please estimate how much carbohydrate you usually consume during a short-duration session (in grams/hour).

As reference, carbohydrate content is: medium banana ~30 g; Lucozade sport drink ~30 g; Mars bar ~38 g; standard energy gel ~20 g.

- 20 grams per hour (7)
- 30 grams per hour (1)
- 60 grams per hour (2)
- 90 grams per hour (3)
- > 90 grams per hour (4)
- I don't know (6)

Display This Question:

If The following statements will ask you about your practices around carbohydrate intake for the dif... = For a virtual racing event shorter than 1h, I intentionally consume carbohydrates: [ After ]

Q75 You indicated that you intentionally consume carbohydrates *after* a short-duration (<1h) racing event. Please estimate how much carbohydrate you usually consume after a short-duration session (in grams).

As reference, carbohydrate content is: medium banana ~30 g; Lucozade sport drink ~30 g; Mars bar ~38 g; standard energy gel ~20 g.

- 20 grams (7)
- 30 grams (1)
- 60 grams (2)
- 90 grams (3)
- > 90 grams (4)
- I don't know (6)

Display This Question:

If The following statements will ask you about your practices around carbohydrate intake for the dif... = For a structured work-out session shorter than 1h, I intentionally consume carbohydrates: [ After ]

Q76 You indicated that you intentionally consume carbohydrates *after* a short-duration (<1h) structured work-out. Please estimate how much carbohydrate you usually consume after a short-duration session (in grams).

As reference, carbohydrate content is: medium banana ~30 g; Lucozade sport drink ~30 g; Mars bar ~38 g; standard energy gel ~20 g.

- 20 grams (7)
- 30 grams (1)
- 60 grams (2)
- 90 grams (3)
- > 90 grams (4)
- I don't know (6)

Display This Question:

If The following statements will ask you about your practices around carbohydrate intake for the dif... = For an unstructured session shorter than 1h, I intentionally consume carbohydrates: [ After ]

Q77 You indicated that you intentionally consume carbohydrates *after* a short-duration (<1h) unstructured session. Please estimate how much carbohydrate you usually consume after a short-duration session (in grams).

As reference, carbohydrate content is: medium banana ~30 g; Lucozade sport drink ~30 g; Mars bar ~38 g; standard energy gel ~20 g.

- 20 grams (7)
- 30 grams (1)
- 60 grams (2)
- 90 grams (3)
- > 90 grams (4)
- I don't know (6)

Display This Question:

If The following statements will ask you about your practices around carbohydrate intake for the dif... = For a virtual racing event longer than 1h, I intentionally consume carbohydrates: [ Before ]

Q78 You indicated that you intentionally consume carbohydrates *before* a long-duration (>1h) racing event. Please estimate how much carbohydrate you usually consume before a long-duration session (in grams).

 As reference, carbohydrate content is: medium banana ~30 g; Lucozade sport drink ~30 g; Mars bar ~38 g; standard energy gel ~20 g.

- 20 grams (7)
- 30 grams (1)
- 60 grams (2)
- 90 grams (3)
- > 90 grams (4)
- I don't know (6)

Display This Question:

If The following statements will ask you about your practices around carbohydrate intake for the dif... = For a structured work-out session longer than 1h, I intentionally consume carbohydrates: [ Before ]

Q79 You indicated that you intentionally consume carbohydrates *before* a long-duration (>1h) structured work-out. Please estimate how much carbohydrate you usually consume before a long-duration session (in grams).

 As reference, carbohydrate content is: medium banana ~30 g; Lucozade sport drink ~30 g; Mars bar ~38 g; standard energy gel ~20 g.

- 20 grams (7)
- 30 grams (1)
- 60 grams (2)
- 90 grams (3)
- > 90 grams (4)
- I don't know (6)

Display This Question:

If The following statements will ask you about your practices around carbohydrate intake for the dif... = For an unstructured session longer than 1h, I intentionally consume carbohydrates: [ Before ]

Q80 You indicated that you intentionally consume carbohydrates *before* a long-duration (>1h) unstructured session. Please estimate how much carbohydrate you usually consume before a long-duration session (in grams).

 As reference, carbohydrate content is: medium banana ~30 g; Lucozade sport drink ~30 g; Mars bar ~38 g; standard energy gel ~20 g.

- 20 grams (7)
- 30 grams (1)
- 60 grams (2)
- 90 grams (3)
- > 90 grams (4)
- I don't know (6)

Display This Question:

If The following statements will ask you about your practices around carbohydrate intake for the dif... = For a virtual racing event longer than 1h, I intentionally consume carbohydrates: [ During ]

Q81 You indicated that you intentionally consume carbohydrates *during* a long-duration (>1h) racing event. Please estimate how much carbohydrate you usually consume during a long-duration session (in grams/hour). If the quantity changes across longer durations, select all answers that apply.

As reference, carbohydrate content is: medium banana ~30 g; Lucozade sport drink ~30 g; Mars bar ~38 g; standard energy gel ~20 g.

- 20 grams per hour (7)
- 30 grams per hour (1)
- 60 grams per hour (2)
- 90 grams per hour (3)
- > 90 grams per hour (4)
- I don't know (6)

Display This Question:

If The following statements will ask you about your practices around carbohydrate intake for the dif... = For a structured work-out session longer than 1h, I intentionally consume carbohydrates: [ During ]

Q82 You indicated that you intentionally consume carbohydrates *during* a long-duration (>1h) structured work-out. Please estimate how much carbohydrate you usually consume during a long-duration session (in grams/hour). If the quantity changes across longer durations, select all answers that apply.

As reference, carbohydrate content is: medium banana ~30 g; Lucozade sport drink ~30 g; Mars bar ~38 g; standard energy gel ~20 g.

- 20 grams per hour (7)
- 30 grams per hour (1)
- 60 grams per hour (2)
- 90 grams per hour (3)
- > 90 grams per hour (4)
- I don't know (6)

Display This Question:

If The following statements will ask you about your practices around carbohydrate intake for the dif... = For an unstructured session longer than 1h, I intentionally consume carbohydrates: [ During ]

Q83 You indicated that you intentionally consume carbohydrates *during* a long-duration (>1h) unstructured session. Please estimate how much carbohydrate you usually consume during a long-duration session (in grams/hour). If the quantity changes across longer durations, select all answers that apply.

As reference, carbohydrate content is: medium banana ~30 g; Lucozade sport drink ~30 g; Mars bar ~38 g; standard energy gel ~20 g.

- 20 grams per hour (7)
- 30 grams per hour (1)
- 60 grams per hour (2)
- 90 grams per hour (3)
- > 90 grams per hour (4)
- I don't know (6)

Display This Question:

If The following statements will ask you about your practices around carbohydrate intake for the dif... = For a virtual racing event longer than 1h, I intentionally consume carbohydrates: [ After ]

Q84 You indicated that you intentionally consume carbohydrates *after*a long-duration (>1h) racing event. Please estimate how much carbohydrate you usually consume after a long-duration session (in grams).

As reference, carbohydrate content is: medium banana ~30 g; Lucozade sport drink ~30 g; Mars bar ~38 g; standard energy gel ~20 g.

- 20 grams (7)
- 30 grams (1)
- 60 grams (2)
- 90 grams (3)
- > 90 grams (4)
- I don't know (6)

Display This Question:

If The following statements will ask you about your practices around carbohydrate intake for the dif... = For a structured work-out session longer than 1h, I intentionally consume carbohydrates: [ After ]

Q85 You indicated that you intentionally consume carbohydrates *after*a long-duration (>1h) structured work-out. Please estimate how much carbohydrate you usually consume after a long-duration session (in grams).

As reference, carbohydrate content is: medium banana ~30 g; Lucozade sport drink ~30 g; Mars bar ~38 g; standard energy gel ~20 g.

- 20 grams (7)
- 30 grams (1)
- 60 grams (2)
- 90 grams (3)
- > 90 grams (4)
- I don't know (6)

Display This Question:

If The following statements will ask you about your practices around carbohydrate intake for the dif... = For an unstructured session longer than 1h, I intentionally consume carbohydrates: [ After ]

Q86 You indicated that you intentionally consume carbohydrates *after*a long-duration (>1h) unstructured session. Please estimate how much carbohydrate you usually consume after a long-duration session (in grams).

As reference, carbohydrate content is: medium banana ~30 g; Lucozade sport drink ~30 g; Mars bar ~38 g; standard energy gel ~20 g.

- 20 grams (7)
- 30 grams (1)
- 60 grams (2)
- 90 grams (3)
- > 90 grams (4)
- I don't know (6)

Q87 The following statements will ask you about your practices around ***protein*intake** for the different indoor cycling sessions. Practices will be separated based on type (racing event, structured work-out, unstructured session) and duration (short-duration < 1h, long-duration >1h)

Please indicate whether you *intentionally* consume protein to support your exercise session. Select all time points that apply.

|  | Before (1) | During (2) | After (3) | I don't plan/I don't have a strategy for this event (4) | I have never engaged in such a session (5) |
| --- | --- | --- | --- | --- | --- |
| For a virtual racing event shorter than 1h, I intentionally consume protein: (1) |  |  |  |  |  |
| For a structured work-out session shorter than 1h, I intentionally consume protein: (2) |  |  |  |  |  |
| For an unstructured session shorter than 1h, I intentionally consume protein: (3) |  |  |  |  |  |
| For a virtual racing event longer than 1h, I intentionally consume protein: (5) |  |  |  |  |  |
| For a structured work-out session longer than 1h, I intentionally consume protein: (6) |  |  |  |  |  |
| For an unstructured session longer than 1h, I intentionally consume protein: (7) |  |  |  |  |  |

Display This Question:

If The following statements will ask you about your practices around protein intake for the differen... = For a virtual racing event shorter than 1h, I intentionally consume protein: [ Before ]

Q88 You indicated that you intentionally consume **protein** *before*a short-duration (<1h) racing event. Please estimate how much protein you usually consume before a short-duration session (in grams).

As reference, protein content is: 250g yoghurt ~12 g; 250ml semi-skimmed milk ~10 g; 30g protein powder ~25 g.

- 10 grams (7)
- 20 grams (1)
- 30 grams (2)
- 40 grams (3)
- > 40 grams (4)
- I don't know (6)

Display This Question:

If The following statements will ask you about your practices around protein intake for the differen... = For a structured work-out session shorter than 1h, I intentionally consume protein: [ Before ]

Q89 You indicated that you intentionally consume **protein** *before*a short-duration (<1h) structured work-out. Please estimate how much protein you usually consume before a short-duration session (in grams).

As reference, protein content is: 250g yoghurt ~12 g; 250ml semi-skimmed milk ~10 g; 30g protein powder ~25 g.

- 10 grams (7)
- 20 grams (1)
- 30 grams (2)
- 40 grams (3)
- > 40 grams (4)
- I don't know (6)

Display This Question:

If The following statements will ask you about your practices around protein intake for the differen... = For an unstructured session shorter than 1h, I intentionally consume protein: [ Before ]

Q90 You indicated that you intentionally consume **protein** *before*a short-duration (<1h) unstructured session. Please estimate how much protein you usually consume before a short-duration session (in grams).

As reference, protein content is: 250g yoghurt ~12 g; 250ml semi-skimmed milk ~10 g; 30g protein powder ~25 g.

- 10 grams (7)
- 20 grams (1)
- 30 grams (2)
- 40 grams (3)
- > 40 grams (4)
- I don't know (6)

Display This Question:

If The following statements will ask you about your practices around protein intake for the differen... = For a virtual racing event shorter than 1h, I intentionally consume protein: [ During ]

Q91 You indicated that you intentionally consume **protein** *during*a short-duration (<1h) racing event. Please estimate how much protein you usually consume during a short-duration session (in grams).

As reference, protein content is: 250g yoghurt ~12 g; 250ml semi-skimmed milk ~10 g; 30g protein powder ~25 g.

- 10 grams (7)
- 20 grams (1)
- 30 grams (2)
- 40 grams (3)
- > 40 grams (4)
- I don't know (6)

Display This Question:

If The following statements will ask you about your practices around protein intake for the differen... = For a structured work-out session shorter than 1h, I intentionally consume protein: [ During ]

Q92 You indicated that you intentionally consume **protein** *during*a short-duration (<1h) structured work-out. Please estimate how much protein you usually consume during a short-duration session (in grams).

As reference, protein content is: 250g yoghurt ~12 g; 250ml semi-skimmed milk ~10 g; 30g protein powder ~25 g.

- 10 grams (7)
- 20 grams (1)
- 30 grams (2)
- 40 grams (3)
- > 40 grams (4)
- I don't know (6)

Display This Question:

If The following statements will ask you about your practices around protein intake for the differen... = For an unstructured session shorter than 1h, I intentionally consume protein: [ During ]

Q93 You indicated that you intentionally consume **protein** *during*a short-duration (<1h) unstructured session. Please estimate how much protein you usually consume during a short-duration session (in grams).

As reference, protein content is: 250g yoghurt ~12 g; 250ml semi-skimmed milk ~10 g; 30g protein powder ~25 g.

- 10 grams (7)
- 20 grams (1)
- 30 grams (2)
- 40 grams (3)
- > 40 grams (4)
- I don't know (6)

Display This Question:

If The following statements will ask you about your practices around protein intake for the differen... = For a virtual racing event shorter than 1h, I intentionally consume protein: [ After ]

Q94 You indicated that you intentionally consume **protein** *after*a short-duration (<1h) racing event. Please estimate how much protein you usually consume after a short-duration session (in grams).

As reference, protein content is: 250g yoghurt ~12 g; 250ml semi-skimmed milk ~10 g; 30g protein powder ~25 g.

- 10 grams (7)
- 20 grams (1)
- 30 grams (2)
- 40 grams (3)
- > 40 grams (4)
- I don't know (6)

Display This Question:

If The following statements will ask you about your practices around protein intake for the differen... = For a structured work-out session shorter than 1h, I intentionally consume protein: [ After ]

Q95 You indicated that you intentionally consume **protein** *after*a short-duration (<1h) structured work-out. Please estimate how much protein you usually consume after a short-duration session (in grams).

As reference, protein content is: 250g yoghurt ~12 g; 250ml semi-skimmed milk ~10 g; 30g protein powder ~25 g.

- 10 grams (7)
- 20 grams (1)
- 30 grams (2)
- 40 grams (3)
- > 40 grams (4)
- I don't know (6)

Display This Question:

If The following statements will ask you about your practices around protein intake for the differen... = For an unstructured session shorter than 1h, I intentionally consume protein: [ After ]

Q96 You indicated that you intentionally consume **protein** *after*a short-duration (<1h) unstructured session. Please estimate how much protein you usually consume after a short-duration session (in grams).

As reference, protein content is: 250g yoghurt ~12 g; 250ml semi-skimmed milk ~10 g; 30g protein powder ~25 g.

- 10 grams (7)
- 20 grams (1)
- 30 grams (2)
- 40 grams (3)
- > 40 grams (4)
- I don't know (6)

Display This Question:

If The following statements will ask you about your practices around protein intake for the differen... = For a virtual racing event longer than 1h, I intentionally consume protein: [ Before ]

Q97 You indicated that you intentionally consume **protein** *before*a long-duration (>1h) racing event. Please estimate how much protein you usually consume before a long-duration session (in grams).

As reference, protein content is: 250g yoghurt ~12 g; 250ml semi-skimmed milk ~10 g; 30g protein powder ~25 g.

- 10 grams (7)
- 20 grams (1)
- 30 grams (2)
- 40 grams (3)
- > 40 grams (4)
- I don't know (6)

Display This Question:

If The following statements will ask you about your practices around protein intake for the differen... = For a structured work-out session longer than 1h, I intentionally consume protein: [ Before ]

Q98 You indicated that you intentionally consume **protein** *before*a long-duration (>1h) structured work-out. Please estimate how much protein you usually consume before a long-duration session (in grams).

As reference, protein content is: 250g yoghurt ~12 g; 250ml semi-skimmed milk ~10 g; 30g protein powder ~25 g.

- 10 grams (7)
- 20 grams (1)
- 30 grams (2)
- 40 grams (3)
- > 40 grams (4)
- I don't know (6)

Display This Question:

If The following statements will ask you about your practices around protein intake for the differen... = For an unstructured session longer than 1h, I intentionally consume protein: [ Before ]

Q99 You indicated that you intentionally consume **protein** *before*a long-duration (>1h) unstructured session. Please estimate how much protein you usually consume before a long-duration session (in grams).

As reference, protein content is: 250g yoghurt ~12 g; 250ml semi-skimmed milk ~10 g; 30g protein powder ~25 g.

- 10 grams (7)
- 20 grams (1)
- 30 grams (2)
- 40 grams (3)
- > 40 grams (4)
- I don't know (6)

Display This Question:

If The following statements will ask you about your practices around protein intake for the differen... = For a virtual racing event longer than 1h, I intentionally consume protein: [ During ]

Q100 You indicated that you intentionally consume **protein** *during*a long-duration (>1h) racing event. Please estimate how much protein you usually consume during a long-duration session (in grams).

As reference, protein content is: 250g yoghurt ~12 g; 250ml semi-skimmed milk ~10 g; 30g protein powder ~25 g.

- 10 grams (7)
- 20 grams (1)
- 30 grams (2)
- 40 grams (3)
- > 40 grams (4)
- I don't know (6)

Display This Question:

If The following statements will ask you about your practices around protein intake for the differen... = For a structured work-out session longer than 1h, I intentionally consume protein: [ During ]

Q101 You indicated that you intentionally consume **protein** *during*a long-duration (>1h) structured work-out. Please estimate how much protein you usually consume during a long-duration session (in grams).

As reference, protein content is: 250g yoghurt ~12 g; 250ml semi-skimmed milk ~10 g; 30g protein powder ~25 g.

- 10 grams (7)
- 20 grams (1)
- 30 grams (2)
- 40 grams (3)
- > 40 grams (4)
- I don't know (6)

Display This Question:

If The following statements will ask you about your practices around protein intake for the differen... = For an unstructured session longer than 1h, I intentionally consume protein: [ During ]

Q102 You indicated that you intentionally consume **protein** *during*a long-duration (>1h) unstructured session. Please estimate how much protein you usually consume during a long-duration session (in grams).

As reference, protein content is: 250g yoghurt ~12 g; 250ml semi-skimmed milk ~10 g; 30g protein powder ~25 g.

- 10 grams (7)
- 20 grams (1)
- 30 grams (2)
- 40 grams (3)
- > 40 grams (4)
- I don't know (6)

Display This Question:

If The following statements will ask you about your practices around protein intake for the differen... = For a virtual racing event longer than 1h, I intentionally consume protein: [ After ]

Q103 You indicated that you intentionally consume **protein** *after*a long-duration (>1h) racing event. Please estimate how much protein you usually consume before a long-duration session (in grams).

As reference, protein content is: 250g yoghurt ~12 g; 250ml semi-skimmed milk ~10 g; 30g protein powder ~25 g.

- 10 grams (7)
- 20 grams (1)
- 30 grams (2)
- 40 grams (3)
- > 40 grams (4)
- I don't know (6)

Display This Question:

If The following statements will ask you about your practices around protein intake for the differen... = For a structured work-out session longer than 1h, I intentionally consume protein: [ After ]

Q104 You indicated that you intentionally consume **protein** *after*a long-duration (>1h) structured work-out. Please estimate how much protein you usually consume before a long-duration session (in grams).

As reference, protein content is: 250g yoghurt ~12 g; 250ml semi-skimmed milk ~10 g; 30g protein powder ~25 g.

- 10 grams (7)
- 20 grams (1)
- 30 grams (2)
- 40 grams (3)
- > 40 grams (4)
- I don't know (6)

Display This Question:

If The following statements will ask you about your practices around protein intake for the differen... = For an unstructured session longer than 1h, I intentionally consume protein: [ After ]

Q105 You indicated that you intentionally consume **protein** *after*a long-duration (>1h) unstructured session. Please estimate how much protein you usually consume before a long-duration session (in grams).

As reference, protein content is: 250g yoghurt ~12 g; 250ml semi-skimmed milk ~10 g; 30g protein powder ~25 g.

- 10 grams (7)
- 20 grams (1)
- 30 grams (2)
- 40 grams (3)
- > 40 grams (4)
- I don't know (6)

Q106 Which of the following list of supplements below have you ever used with the aim to increase performance or to complete an indoor cycling session? Please select all that apply. Please make sure to tick "I have never used this supplement" if this is the case.

|  | Virtual racing event (1) | Structured work-out (2) | Unstructured (3) | I have never used this supplement (4) |
| --- | --- | --- | --- | --- |
| Beetroot juice/nitrate-rich products (1) |  |  |  |  |
| Beta-alanine (2) |  |  |  |  |
| Caffeine (3) |  |  |  |  |
| Creatine (4) |  |  |  |  |
| Menthol (5) |  |  |  |  |
| Sodium bicarbonate (6) |  |  |  |  |
| Any other (7) |  |  |  |  |

Display This Question:

If Which of the following list of supplements below have you ever used with the aim to increase perf... = Any other [ Virtual racing event ]

Q107 What other supplement have you used to successfully complete a virtual racing event?

________________________________________________________________

Display This Question:

If Which of the following list of supplements below have you ever used with the aim to increase perf... = Any other [ Structured work-out ]

Q108 What other supplement have you used to successfully complete a structured work-out?

________________________________________________________________

Display This Question:

If Which of the following list of supplements below have you ever used with the aim to increase perf... = Any other [ Unstructured ]

Q109 What other supplement have you used to successfully complete an unstructured cycling session?

________________________________________________________________

Q110 Are there any other nutritional strategies that you deliberately and typically use for either short or long-duration indoor cycling sessions that have not been covered by the questionnaire? Examples could be: fasted exercise or following a ketogenic low-carbohydrate high-fat diet.

Please use the text entry to describe this strategy. If this is not the case, you can move to the next question and leave this section blank.

________________________________________________________________

________________________________________________________________

________________________________________________________________

________________________________________________________________

________________________________________________________________

End of Block: Nutrition
